# Cellular senescence with SASP in periodontal ligament cells triggers inflammation in aging periodontal tissue

**DOI:** 10.18632/aging.204569

**Published:** 2023-03-01

**Authors:** Kuniko Ikegami, Motozo Yamashita, Mio Suzuki, Tomomi Nakamura, Koki Hashimoto, Jirouta Kitagaki, Manabu Yanagita, Masahiro Kitamura, Shinya Murakami

**Affiliations:** 1 Department of Periodontology, Graduate School of Dentistry, Osaka University, Suita, Osaka 565-0871, Japan

**Keywords:** cellular senescence, periodontitis, periodontal ligament, SASP, microRNAs, SIRT1

## Abstract

The direct cause of periodontitis is periodontopathic bacteria, while various environmental factors affect the severity of periodontitis. Previous epidemiological studies have shown positive correlations between aging and periodontitis. However, whether and how aging is linked to periodontal health and disease in biological processes is poorly understood. Aging induces pathological alterations in organs, which promotes systemic senescence associated with age-related disease. Recently, it has become evident that senescence at the cellular level, cellular senescence, is a cause of chronic diseases through production of various secretory factors including proinflammatory cytokines, chemokines, and matrix metalloproteinases (MMPs), which is referred to the senescence-associated secretory phenotype (SASP). In this study, we examined the pathological roles of cellular senescence in periodontitis. We found localization of senescent cells in periodontal tissue, particularly the periodontal ligament (PDL), in aged mice. Senescent human PDL (HPDL) cells showed irreversible cell cycle arrest and SASP-like phenotypes *in vitro*. Additionally, we observed age-dependent upregulation of microRNA (miR)-34a in HPDL cells. These results suggest that chronic periodontitis is mediated by senescent PDL cells that exacerbate inflammation and destruction of periodontal tissues through production of SASP proteins. Thus, miR-34a and senescent PDL cells might be promising therapeutic targets for periodontitis in elderly people.

## INTRODUCTION

Periodontitis is a chronic inflammatory disease characterized by periodontal tissue destruction with loss of tooth-supportive bone. It is thought to be the most common infectious disease and affects more than 40% of people aged over 30 years [1]. Colonization of dental biofilm involving periodontopathic bacteria triggers inflammation and excessive immune responses that exacerbate breakdown of periodontal tissue. In addition to bactericidal pathogens, various environmental factors affect the pathology and progression of periodontal disease. In particular, aging has been recognized as a major risk factor that affects the onset and severity of periodontitis [2]. Thus, understanding the biological mechanisms that regulate periodontal tissue and health by aging is an urgent issue to establish preventive protocols or specialized therapies for elderly persons in the field of periodontal medicine.

In the process of aging, accumulated environmental stresses induce degeneration of multiple organs, which accelerates the morbidity and severity of lifestyle diseases [3]. Numerous epidemiological studies and diseased animal models have shown positive correlations between periodontitis and age-dependent, lifestyle-related diseases such as type 2 diabetes, obesity, rheumatoid arthritis, and heart infarction [4, 5]. Therefore, periodontitis and these lifestyle-related diseases would share common pathology for disease development. Previous attempts to clarify the effects of aging on homeostasis of periodontal tissue are limited [6–8] and periodontal diseases have not been fully elucidated at the molecular level.

Cellular senescence is a major hallmark of senescence in organs and the whole body. Accumulated senescent cells in aged organs and tissues induce senescence of the body [9]. Cellular senescence is defined as a state of irreversible cell cycle arrest, but not apoptosis, in mammalian cells. Initially, cellular senescence was thought to be an intrinsic cellular mechanism to escape tumorigenesis. A large number of studies have indicated that senescent cells secrete various proteins such as proinflammatory cytokines, chemokines, growth factors, and metalloproteinases, termed SASP (senescence-associated secretory proteins) [10]. First, SASP was considered to be the prominent mechanism for recruitment of immunocompetent cells to eliminate tumor cells in aged organs. Recent studies have shown that senescent cells induce inflammation and impair wound healing in various chronic diseases, such as rheumatoid arthritis, atherosclerosis, and osteoporosis, through induction of SASP [5, 11]. Therefore, understanding cellular senescence is required to develop more effective therapies and prevention protocols for age-dependent, lifestyle-related diseases. However, whether and how cell types within periodontal tissue undergo cellular senescence with SASP have not yet been clarified.

Periodontal ligament (PDL) is a soft fibrous tissue located between the tooth cementum and alveolar bone. PDL cells produce extracellular matrix (ECM) proteins, such as type I/III collagens and fibronectin, to maintain physiological elasticity and manage the mechanistic occlusal force in PDL [12]. PDL not only physically supports teeth, but also plays many biological roles in periodontal tissue. For example, PDL cells are responsible for local immune responses by producing various cytokines and adhesion molecules to act as a biological barrier in orchestration with other cells in periodontal tissue [13]. Additionally, multipotent mesenchymal stem cells in PDL tissue proliferate or differentiate for wound repair and tissue regeneration [14, 15]. Therefore, maintaining homeostasis in PDL is important for periodontal tissue. Impaired PDL is thought to be a risk to periodontal health, since defects in PDL at cellular levels can trigger the breakdown of periodontal tissue, especially in aged people.

microRNAs (miRNAs), a class of small non-coding RNAs expressed in eukaryotes, are 20–22 nucleotide, endogenous single-stranded RNAs [16]. miRNAs suppress gene expression by inhibiting translation of their target genes and degrading target mRNAs through binding to complementary sites located in the 3′-untranslated regions of target mRNAs in a highly context-dependent manner. More than 2000 miRNAs have been identified and each miRNA can target hundreds of genes on basis of their short sequence. Thus, miRNAs are proposed to regulate various biological processes such as development, tumorigenesis, and organism aging through modulation of inflammation and cellular senescence [17–19]. In the past two decades, many kinds of miRNAs have been identified as causes of chronic inflammation [20] and many miRNAs target the NF-κB pathway, such as miR-146a/b, miR-155, and miR-21, which are thought to modulate the inflammatory response in various cell types [21–23]. Recent studies have indicated possible relationships between miRNAs and periodontitis [24]. Therefore, identification of senescent HPDL cells and elucidation of their molecular mechanisms, including miRNAs, are required to better understand periodontitis.

In this study, we aimed to clarify the pathophysiological roles of cellular senescence in periodontal tissue and diseases. Identification of the functions of cellular senescence in periodontal tissues may facilitate gaining deep insights into inflammation and destruction of periodontal tissues in elderly people. Development of therapies targeting senescent cells in periodontal tissues may also be an effective strategy for elderly people.

## RESULTS

### Analysis of periodontal tissue in aged mice

**Figure 1:**
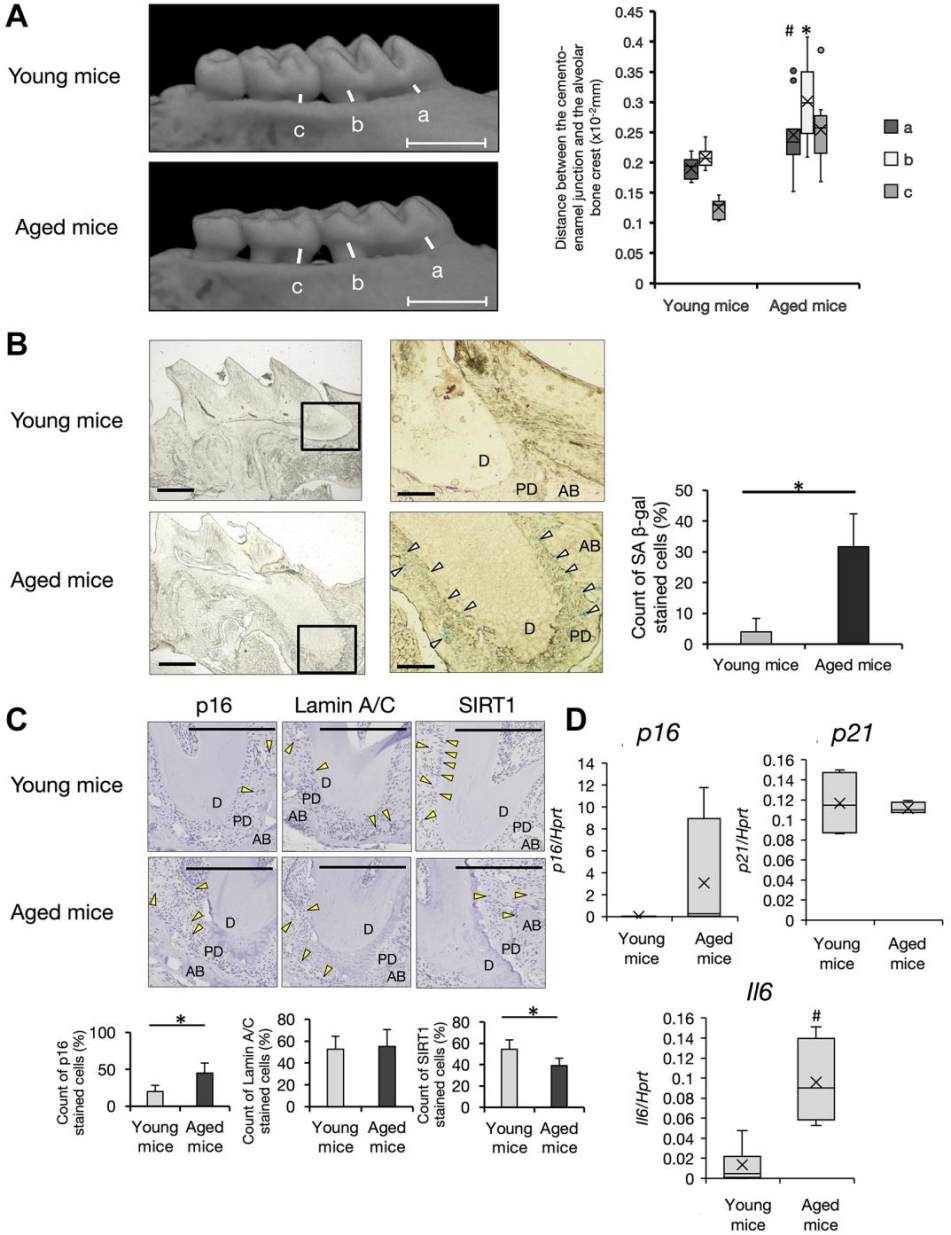
**Analysis of periodontal tissue in aged mice.** (**A**) Micro CT (μCT) analysis of alveolar bone in the upper jaw. Representative images of young (6-week-old) and aged (68~104 weeks-old) mice are shown. Scale bar = 1 mm. Quantification of the bone resorption rate in supportive alveolar bone was evaluated. Distance between the cement–enamel junction to the crest of alveolars bone at the a, mesial root at first molars, b, distal root at first molars, and c, mesial root at second molars. Box-and-whiskers plots shows median, 25th and 75th percentile with whiskers at the 5th and 95th percentile. Young mice; *n* = 7, aged mice; *n* = 11 Statistical analysis was completed using welch’s *t*-test, with *p*-values < 0.05 were considered statistically significant (^*^p < 0.01, ^#^*p* < 0.05). (**B**) X-gal staining of frozen sections of periodontal tissue of the mesial root at first molars in the upper jaw. (×40) White arrows indicate SA-β-gal positive cells. Scale bar = 500 μm. Right panels show the enlarged image of the bold square in the left panel (×100). Scale bar = 100 μm. Abbreviations: D: Dentin; PD: Periodontal ligament; AB: Alveolar Bone. Representative data from three experiments are shown. Right graph shows the percentage of SA-β-gal positive cells in PDL of young or aged mice (^*^p < 0.01). (**C**) Representative immunohistochemistry images of p16, lamin A/C, and SIRT1 (40×). Scale bar = 200 μm. Abbreviations: D: dentin; PD: periodontal ligament; AB: alveolar Bone. Yellow arrow: antibody-positive cells (40×). Representative data from three experiments are shown. Lower graph shows percentages of antibody-positive cells in PDL area of young or aged mice (^*^p < 0.01). (**D**) Expression of p16, p21, and IL-6 in PDL tissue of young (6~13-weeks-old, *n* = 7) and aged (68~104-weeks-old, *n* = 6) mice. Expression of p16, p21, and IL-6 mRNA in PDL derived from freshly isolated mouse teeth was analyzed by qRT-PCR (^#^*p* < 0.05). Representative data from three experiments are shown.

Epidemiological studies have suggested a strong correlation between aging and periodontal diseases [25]. First, we compared alteration of the bone volume in alveolar bone at the maxilla between 6-week-old (young) and 68-week-old (aged) C57BL/6 male mice by μCT analysis. Aged mice showed a decreased level of the alveolar bone crest with a flattened shape of the tooth cusp (Figure 1A). Bone resorption in the horizontal direction was apparent in supportive alveolar bone surrounding molars in aged mice. Analysis of digital images indicated nearly two-fold bone resorption at molars of aged mice compared with young mice (Figure 1A, Box-and-whiskers plots, young mice; *n* = 7, aged mice; *n* = 11). To examine whether cellular senescence was involved in the pathology of periodontal diseases in aged mice, we performed β-galactosidase (β-gal) staining of periodontal tissues that were used for μCT analysis. Because enhanced β-gal activity in lysosomes at pH 6.0, SA (senescence-associated) β-gal is a general characteristic of cellular senescence *in vitro* and *in vivo* [26]. In aged mice, many SA β-gal-positive cells were found in periodontal tissue, but few cells were found in young mice. Interestingly, SA β-gal-positive cells were mainly localized in the periodontium, but not at gingival connective tissues in aged mice (Figure 1B, Right graph). High magnification images indicated that periodontal ligament cells and endothelial cells around blood vessels were positive for SA β-gal at PDL in aged mice. To confirm this, we performed immunohistochemistry for senescent markers p16 [27], lamin A/C [28], and sirtuin 1 (SIRT1) [29]. In mice aged over 100 weeks, p16 expression was increased (*p* < 0.01) and SIRT1 expression was significantly decreased in periodontium (*p* < 0.01) (Figure 1C). Lamin A/C expression was not changed. Consistent with these findings, p16 mRNA expression was increased in PDL tissue dissected from aged mice. Intriguingly, significant upregulation of the pro-inflammatory cytokine interleukin (IL)-6 was observed in PDL of aged mice (*p* < 0.05) (Figure 1D, young mice; *n* = 7, aged mice; *n* = 6). Thus, senescent cells had accumulated in the periodontium, which may induce inflammation to trigger the breakdown of alveolar bone in aged PDL tissue.

### Establishment of a senescent model of human PDL (HPDL) cells

**Figure 2:**
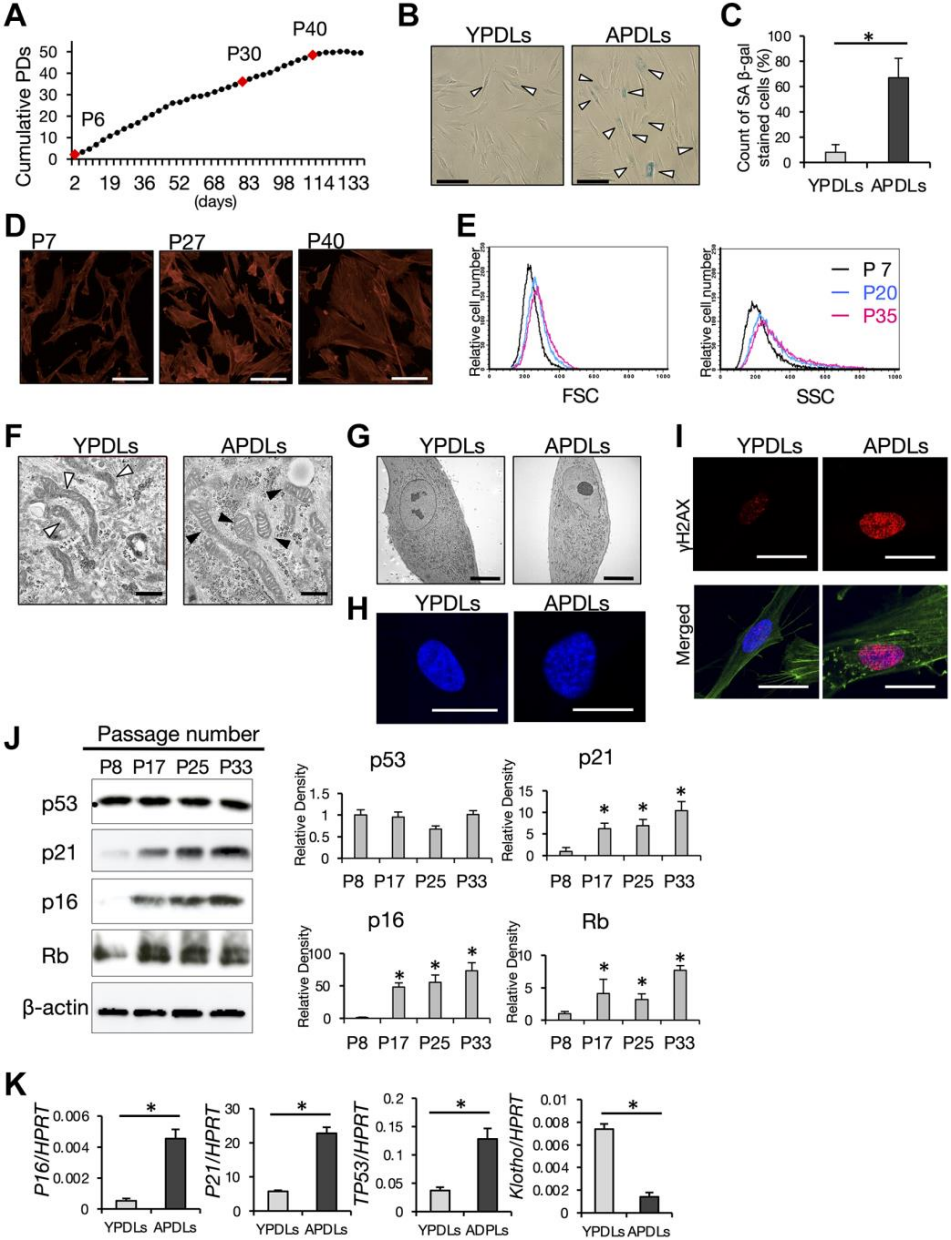
**Establishment of senescent HPDL cells *in vitro*.** (**A**) Long-term growth curve of primary human periodontal ligament (HPDL) cells. Cumulative population doublings (PDs) in each cell passage were estimated in long-term cultures. Final numbers of HPDL cells at the indicated passage are shown. P6, P30, and P40 represent early, premature, and late senescence of HPDL cells *in vitro*. Representative data from three experiments are shown. (**B**) SA β-gal staining of YPDLs and APDLs. Scale bar = 50 μm. White bar: YPDLs; black bar: APDLs (**C**) Quantification of SA β-gal-positive YPDLs and APDLs (^*^p < 0.01). Representative data from three experiments are shown. (**D**) Phalloidin staining of P7, P27, and P40 HPDL cells (×400). Scale bar = 200 μm. Representative data from three experiments are shown. (**E**) Quantification of the size of HPDL cells at P7, P20, and P35 HPDL cells. FSC and SSC of flow cytometric analysis are shown. Representative data from three experiments are shown. (**F**) Representative transmission electron microscopy images of mitochondria in YPDLs and APDLs. White arrows indicate lamellar shaped mitochondria. Black arrows indicate disorganized mitochondria (×31800). Scale bar = 500 nm (**G**) Transmission electron microscopy of induction of aggregated chromosomal DNA in YPDLs and APDLs. (×1760). Scale bar = 10 μm. (**H**) Analysis of SAHF in YPDLs and APDLs. DAPI staining of YPDLs and APDLs (×1000). Scale bar = 25 μm. (**I**) Confocal image of γH2AX staining in YPDLs and APDLs. Red: γH2AX; Green: Actin fiber; Blue: DAPI staining (×400). Scale bar = 25 μm. (**J**) Protein expression of cell cycle arrest-related factors p53, p21, p16, and Rb in P8, P17, P25, and P33 HPDL cells. β-actin was used as a loading control. Representative band images are shown, and the relative protein levels were quantified (^*^p < 0.01). (**K**) Increased expression of senescence-related biomarkers in YPDLs and APDLs. Relative mRNA expression of p16, p21, p53, and klotho to HPRT in HPDL cells quantified by qRT-PCR. Gray bar: YPDLs; Black bar: APDLs. Data are presented as the mean ± SE (^*^p < 0.01). Representative data from three experiments are shown.

Molecular mechanisms underlying biological alterations and degeneration of tissue and organs with aging are gradually becoming clear. Among these, cellular senescence has emerged as a precise event in tissues and organs induced by aging, and has been recognized as a major cause of senescence in the body. To understand the characteristics of senescent cells in PDL, we applied replicative senescence to primary human PDL (HPDL) cells *in vitro*. Repetition of serial passaging is a general method to induce cellular senescence in cultured cells by cellular replication [30]. Somatic HPDL cells showed a maximum of around 40 population doublings (PDs) *in vitro* (Figure 2A). In accordance with the progression of passaging, broad, enlarged, and flattened morphological changes were apparent in HPDL cells during the cultivation period (Figure 2D). The growth rate of HPDL cells was reduced gradually and then the proliferative capacity almost reached its limit at around P35 that corresponded to 45 PDs (Figure 2A). HPDL cells at >P40 showed irreversible cell growth arrest defined as stable cell cycle exit, but not cell death. Attenuation of the growth rate in HPDL cells indicated induction of cellular senescence in a physiological manner (Figure 2A).

To confirm cellular senescence of HPDL cells *in vitro*, we examined SA β-gal activity in HPDL cells (Figure 2B). Around 70% of aged HPDL cells (APDLs, >P30) were positive for SA β-gal, where <10% of young HPDL cells (YPDLs, < P10) were positive for SA β-gal (Figure 2C). To characterize the morphological changes of HPDL cells, phalloidin staining was performed to visualize actin stress fibers. P7 HPDL cells clearly demonstrated a spindle-like cell shape with a compacted cell size, whereas P40 HPDL cells showed an enlarged cell shape with a spread shape (Figure 2D). FCM analysis confirmed the increase in cell size (FSC) and granularity (SSC) of APDLs compared with YPDLs at the single cell level (Figure 2E).

### Senescent HPDL cells produce ROS

Senescent cells partly indicate metabolic changes such as impaired energy metabolism, autophagy, and glycolysis [31, 32]. To examine such changes, we observed the anatomical features of mitochondria, the major ATP/ADP power producers, by TEM analysis. Most mitochondria in APDLs showed a shortened bulge-like shape that was quite different from that of YPDLs, mitochondrial cristae in APDLs had a disrupted structure with ladder-like repeats in the short axis, whereas mitochondrial cristae in YPDLs had a normal structure with a longitudinal elongated shape (Figure 2F). Irregularly shaped mitochondria suggest damage, which produces excess ROS with failure of the redox balance. Consistent with the above observations, CM-H2CDFDA, which is a cell-permeable ROS indicator, stained accumulated cytosolic ROS in APDLs more strongly than in YPDLs (Supplementary Figure 1A, 1B).

### Dysregulation of the chromatin structure in senescent HPDL cells

Robust compaction of chromosomes in the nucleus indicates heterochromatin formation [33]. Because alteration of the epigenetic landscape is a hallmark of cellular senescence in aging, we examined formation of senescence associated heterochromatin foci (SAHF) in HPDL cells. APDLs showed SAHF with apparent chromatin aggregation recognized by TEM analysis, which were hardly stained by DAPI (Figure 2G, 2H). Furthermore, DNA damage marker histone protein γH2AX was increased in APDLs (Figure 2I).

### Expression of cell cycle regulator proteins is enhanced in senescent HPDL cells

To gain molecular insights into the irreversible cell cycle arrest of senescent HPDL cells, we evaluated the expression of major cell cycle regulators, namely p53, p16, p21, and Rb. Two major pathways – p53-p21 and p16^Ink4a^-RB effector pathways – contribute to cell cycle arrest at G1/S phase through inhibition of CDK2 or CDK4/6 [27]. Protein expression levels of p53, p16, p21, and Rb were increased with the progression of cell replication in HPDL cells (Figure 2J). APDLs showed increased mRNA expression of P21, P16 and p53, whereas Klotho expression was decreased compared with YPDLs (Figure 2K). These data suggest that APDLs, which was induced by >30 serial passages *in vitro*, satisfied the general characteristics of senescent cells.

### Senescent HPDL cells produce SASP proteins

**Figure 3:**
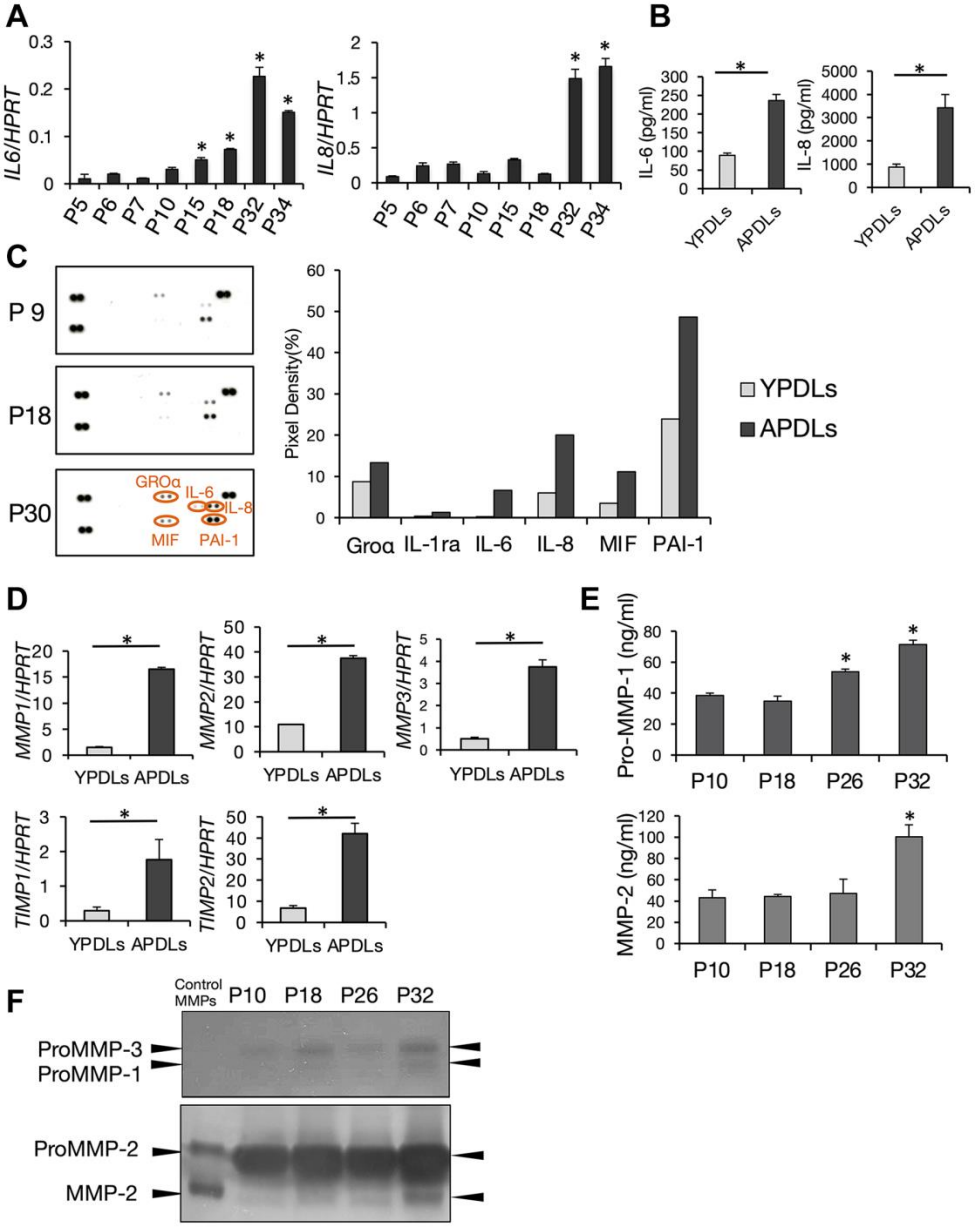
**Increased expression of IL-6 and IL-8 in senescent HPDL cells.** (**A**) Relative mRNA expression of IL-6 and IL-8 in various passages of HPDL cells quantified by qRT-PCR (^*^p < 0.01 vs. P5). (**B**) IL-6 and IL-8 in conditioned medium in YPDLs and APDLs (^*^p < 0.01). (**C**) Enhanced production of SASP factors in senescent HPDL cells. Soluble factors secreted by P9, P18, and P30 HPDL cells were detected by an antibody dot blot array. In right panels, quantification of signal intensity of dots plots assay for conditioned medium of YPDLs and APDLs. Signal intensities of the major dot blots were normalized against control spots in each blot and shown as bar graphs (Groa, IL-1ra, IL-6, IL-8, MIF, PAI-1). Gray bars indicate YPDLs (P9) and black bars indicate APDLs (P30). Representative data from three experiments are shown. (**D**) Relative mRNA expression of MMP-1–3 and TIMP-1 and -2 in HPDL cells quantified by qRT-PCR (^*^p < 0.01). (**E**) Pro-MMP-1 and MMP-2 in conditioned medium of P10, P18, P26, and P32 HPDL cells (^*^p < 0.01 vs. P10). (**F**) Inverted images of zymography for conditioned medium of P10, P18, P26, and P32 HPDL cells. Dark spots indicate Pro-MMP-1–3 and MMP-2. Representative data from three experiments are shown.

Recent studies have revealed a notable function of senescent cells, namely that they secrete various proinflammatory cytokines termed SASP, which affect their neighboring cells. SASP has been reported in various cell types such as fibroblasts [3], epithelial cells [3], vascular endothelial cells [34], and immunocompetent cells [35], and are considered to induce age-dependent inflammation and tissue degeneration in organs. To examine SASP in HPDL cells, we evaluated expression of IL-6 and IL-8 that are major constituents of SASP. Expression levels of IL-6 and IL-8 mRNAs in >P30 HPDL cells were higher than those in early passaged HPDL cells. Expression of IL-6 and IL-8 was increased with progression of cell replication in HPDL cells. Moreover, high production of IL-6 and IL-8 proteins in APDLs was confirmed by ELISAs of culture supernatants (Figure 3A, 3B). Additionally, we performed an antibody-captured cytokine array to monitor highly secreted cytokines other than IL-6 and Il-8 in senescent HPDL cells. As a result, in addition to IL-6 and IL-8, we found that CXCL1, GRO (growth-regulated oncogene)-α, MIF (macrophage migration inhibitory factor) and PAI-1 (plasminogen activator inhibitor-1) in APDLs (P30) were higher than those in YPDLs (P12) (Figure 3C). Density analysis of each spot indicated significant increases in APDLs (right panels, Figure 3C). Moreover, we examined expression of matrix metalloproteinases (MMPs) in senescent HPDL cells. Previous studies have demonstrated enhanced production of MMPs from senescent fibroblasts [36]. mRNA expression of MMP-1–3 and tissue inhibitor of MMPs (TIMP)-1 and 2 in APDLs was significantly higher than that in YPDLs (Figure 3D). Analyses of pro-MMP-1 and MMP-2 protein by ELISAs supported these results (Figure 3E). Furthermore, zymography confirmed enhanced enzymatic activity of pro-MMP-1, pro-MMP-3, pro-MMP-2, and MMP-2 in culture supernatants of APDLs (Figure 3F). Thus, senescent HPDL cells produced various SASP factors, including inflammatory cytokines, chemokines, and MMPs/TIMPs, which affect chronic inflammation.

### Sterile inflammatory phenotype of senescent HPDL cells

**Figure 4:**
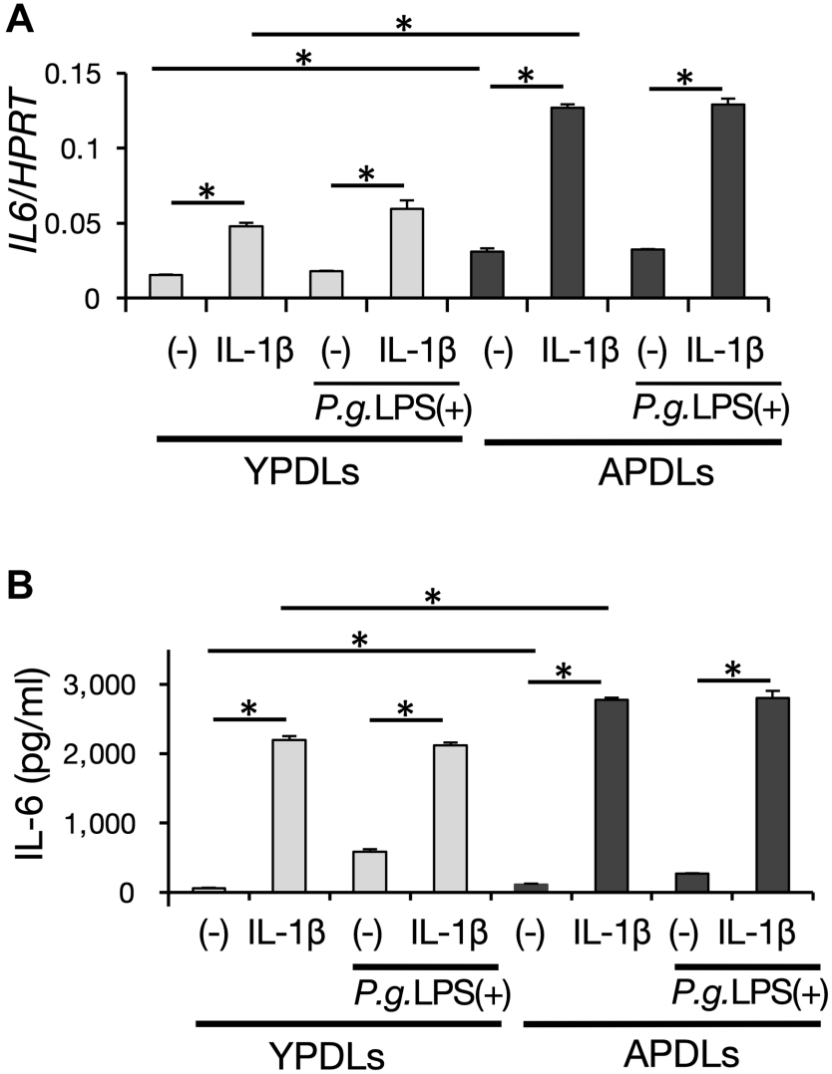
**IL-6 production induced by proinflammatory cytokines and bacterial pathogens in senescent HPDL cells.** (**A**) Relative mRNA expression of IL-6 stimulated by IL-1β (1 ng/ml) and *P.g* LPS (1 μg/ml) in YPDLs and APDLs quantified by qRT-PCR (^*^p < 0.01). (**B**) IL-6 and IL-8 in conditioned medium of YPDLs and APDLs quantified by ELISA (^*^p < 0.01). Representative data from three experiments are shown.

Inflammation in aged organs is partially characterized as sterile inflammation evoked without apparent infection by pathogens [37]. To determine whether this occurred in periodontitis of aged individuals, we examined the inflammatory response in senescent HPDL cells with or without bactericidal stimulation. APDLs showed upregulation of IL-6 mRNA expression in the steady state as described above. Notably, *Porphyromonas gingivalis* (*P.g.*) LPS, which is the major periodontopathic bactericidal pathogen, did not significantly enhance IL-6 mRNA expression; although, the inflammatory cytokine IL-1β induced IL-6 expression in HPDL cells *in vitro*. Moreover, *P.g.* LPS combined with IL-1β stimulation did not enhance IL-6 expression in our system (Figure 4A, Supplementary Figure 2). We confirmed this finding at the protein level by ELISA (Figure 4B). These results suggest that the intrinsic inflammation state of APDLs is higher than YPDLs and susceptibility to bactericidal pathogens but not inflammatory cytokine is low in APDLs.

### Expression of microRNAs in senescent HPDL cells

To clarify the molecular mechanism regulating cellular senescence of HPDL cells, we focused on miRNAs. To date, the roles of miRNAs in senescent characteristics of HPDL cells have not been clarified well. First, we performed comprehensive analysis of miRNAs in HPDL cells by comparing the miRNA expression profiles of HPDL cells at P5, 6, 7, 10, 15, 18, 32, and 34. We found that around 360 miRNAs among 2000 human miRNAs were significantly expressed in HPDL cells. Hierarchical analysis showed that HPDL cells with a close passage number shared similar miRNA expression profiles (Supplementary Figure 3A). This result indicated that replicative senescence of HPDL cells induced by our protocol was appropriate. To classify the variations in miRNA expression profiles during the process of cellular senescence, we performed *K*-means clustering analysis. Among the classified eight patterns, we focused on the miRNA group that increased along with passaging (Supplementary Figure 3B). In this group, seven miRNAs had >2-fold increased expression in P34 HPDL cells (Table 1). Ingenuity pathway analysis (IPA) revealed some miRNAs such as miR-146a and miR-34a that target the inflammation pathway in aged HPDL cells (Supplementary Figures 4 and 5).

**Table 1:** Upregulated expression of miRNAs in senescent HPDL cells.

**miRNA**	**P34/P5 (log2)**
miR-137	7.6
miR-146a	7.2
miR-181a-5p	2.0
miR-181a-1-3p	1.5
miR-34a	1.2
miR-2682	1.1
miR-127	1.1
miR-329	1.1

### Negative regulation of IL-6 by miR-146a in senescent HPDL cells

First, we compared expression of miR-146a using of miRNA array datasets and then performed validation by qRT-PCR (Supplementary Figure 6A). The peak of miR-146a expression in each passage of HPDL cells was delayed compared with the peak of IL-6 expression (left graph, Supplementary Figure 6A). Consistent with a previous study, miR-146a may regulate the senescence phenotype of HPDL cells by silencing IL-6 expression through a negative regulatory mechanism in inflammation of senescent HPDL cells. To analyze the miR-146a function in HPDL cells, we introduced synthetic mimic or inhibitor oligos for miR-146a into HPDL cells (Supplementary Figure 6B). The miR-146a mimic inhibited protein but not mRNA expression of IL-6 in both YPDLs and APDLs. In contrast, effect of the miR-146a inhibitor was not concordant.

### Induction of IL-6 through suppression of SIRT1 by miR-34a in senescent HPDL cells

**Figure 5:**
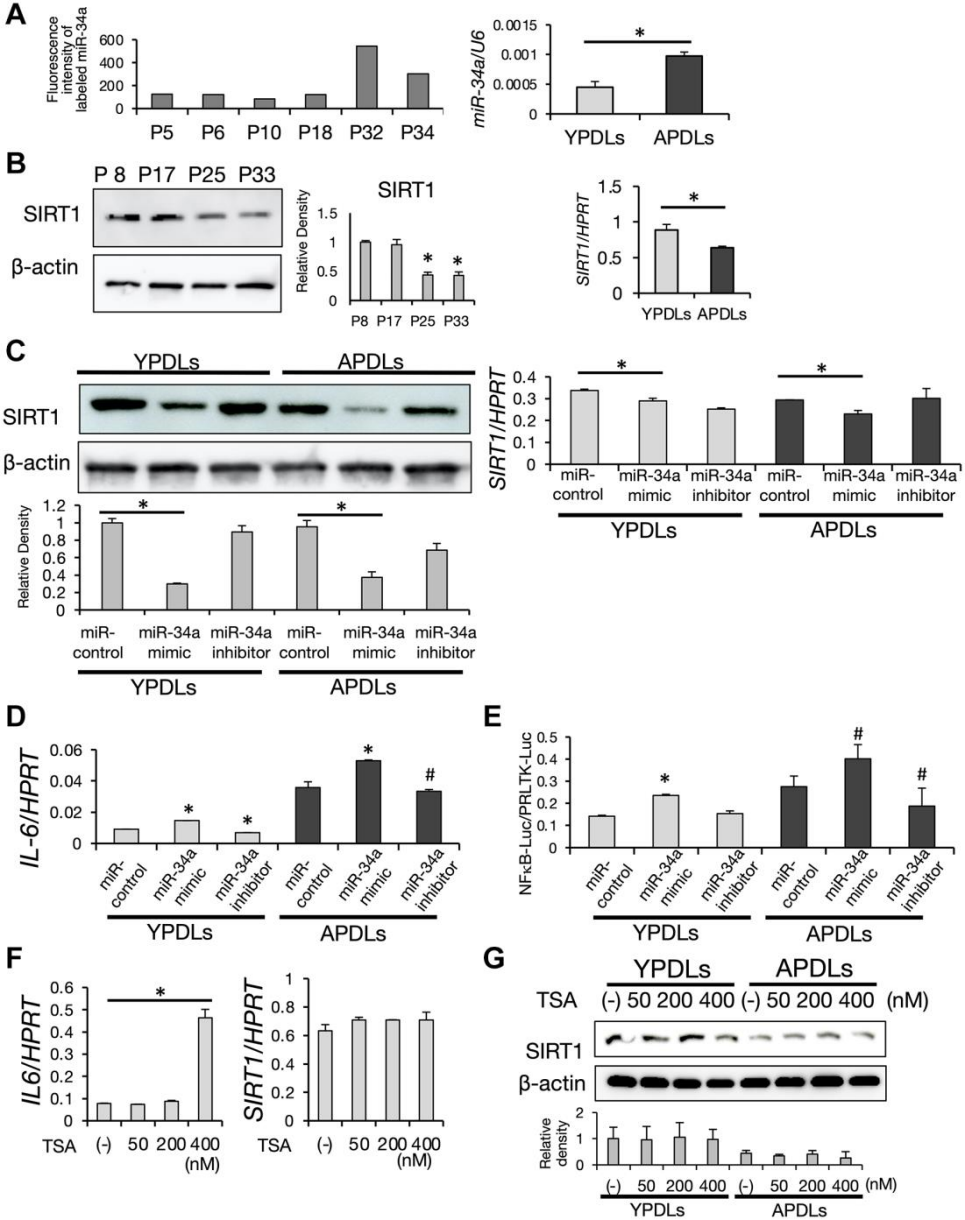
**Increased expression of miR-34a in senescent HPDL cells.** (**A**) Expression of miR-34a was increased depending on the passage of HPDL cells. Scores of the fluorescence intensity of labeled miR-34a in miRNA array analysis of P5, P6, P10, P18, P32, and P34 HPDL cells is displayed in the histogram. Right graph shows the expression of miR-34a in YPDLs (P6) and APDLs (P34) analyzed by qRT-PCR (^*^p < 0.01). (**B**) Decreased expression of SIRT1 in senescent HPDL cells. Expression of SIRT1 protein in P8, P17, P25, and P33 HPDL cells analyzed by western blotting. β-Actin was used as a loading control and the relative protein levels were quantified. (^*^p < 0.01 vs. P8) Right graph shows the expression of SIRT1 mRNA in YPDLs and APDLs measured by qRT-PCR (^*^p < 0.01). (**C**) Overexpression of miR-34a inhibited SIRT1 expression in HPDL cells. MiR-34a mimic and anti-miR-34a oligonucleotides were transfected into YPDLs and APDLs. Expression of SIRT1 protein was analyzed by western blotting. β-Actin was used as a loading control and the relative protein levels were quantified. Right graph shows the expression of SIRT1 measured by qRT-PCR. (^*^p < 0.01 vs. control) (**D**) Overexpression of miR-34a upregulated IL-6 expression in HPDL cells. Expression of IL-6 was measured by qRT-PCR (^*^p < 0.01, ^#^*p* < 0.05 vs. control). (**E**) Overexpression of miR-34a upregulated NF-κB activity in HPDL cells. NF-κB transcription activity was analyzed by a luciferase reporter assay (^*^p < 0.01, ^#^*P* < 0.05 vs. control). (**F**) TSA treatment induced IL-6 in YPDLs (P6). Expression of IL-6 and SIRT1 mRNA in YPDLs after TSA treatment (0, 50, 200, and 400 nM) was quantified by qRT-PCR (^*^p < 0.01 vs. none). (**G**) Expression of SIRT1 in YPDLs and APDLs after TSA treatment (0, 50, 200, and 400 nM). Expression of SIRT1 protein was analyzed by western blotting. β-Actin was used as a loading control and the relative protein levels were quantified. Representative data from three experiments are shown.

The induction mechanism of robust IL-6 production in senescent HPDL cells could not be fully explained by miR-146a alone. Therefore, we examined the role of miR-34a in senescent HPDL cells. As shown in Figure 5A, the expression level of miR-34a was increased dramatically in P32 HPDL cells in the miRNA array dataset, which was confirmed by qRT-PCR analysis. Intriguingly, protein and mRNA expression levels of SIRT1 were sharply decreased in APDLs (Figure 5B). SIRT1 is a homolog of the *Saccharomyces cerevisiae*, *Sir2* protein, a member of the sirtuin family [38], which promotes longevity in many organisms [29, 39]. It has been reported that miR-34a regulates expression of SIRT1 [40]. Introduction of the miR-34a mimic into HPDL cells attenuated expression of SIRT1 (Figure 5C). Additionally, the miR-34a inhibitor rescued SIRT1 expression and suppressed IL-6 expression in APDLs (Figure 5D). These results suggested that the high secretion of IL-6 from senescent HPDL cells was induced by miR-34a through suppression of SIRT1.

### Enhanced NF-κB activity in senescent HPDL cells

SIRT1 is a nicotinamide adenine dinucleotide-dependent class 3 histone deacetylase (HDAC). SIRTs deacetylate the lysine residue in the histone tail of various target genes and cellular proteins [41, 42]. Thus, we hypothesized that miR-34a may regulate IL-6 transcription in senescent HPDL cells via SIRT1 through its HDAC activity. To test this hypothesis, we measured transcriptional activity of endogenous nuclear factor-κB (NF-κB), which plays an important role in transcription of IL-6, in HPDL cells by a reporter assay. As shown in Figure 5E, APDLs had intrinsic high NF-κB activity. MiR-34a mimic treatment induced high NF-κB activity in YPDLs. In contrast, miR-34a inhibitor treatment significantly suppressed NF-κB activity in APDLs. These results suggested that miR-34a induced the transcription of IL-6 through regulation of NF-κB activity. Moreover, we found that trichostatin A (TSA) at 400 nM treatment induced IL-6 expression in YPDL cells without affecting protein expression of SIRT1 (Figure 5F, 5G). TSA is an anti-fungal antibiotic isolated from *Streptomyces hygroscopicus* and reported as a specific inhibitor of histone deacetylases (HDACs) in mammalian cells. TSA selectively inhibits enzymatic activities of class 1 and 2 HDACs, but not class 3 HDAC SIRT1 [43]. Therefore, our results suggest that IL-6 production in HPDL cells was induced by either SIRT1-dependent or -independent mechanisms of histone acetylation.

### SIRT1 suppresses IL-6 in an epigenetic manner in senescent HPDL cells

**Figure 6:**
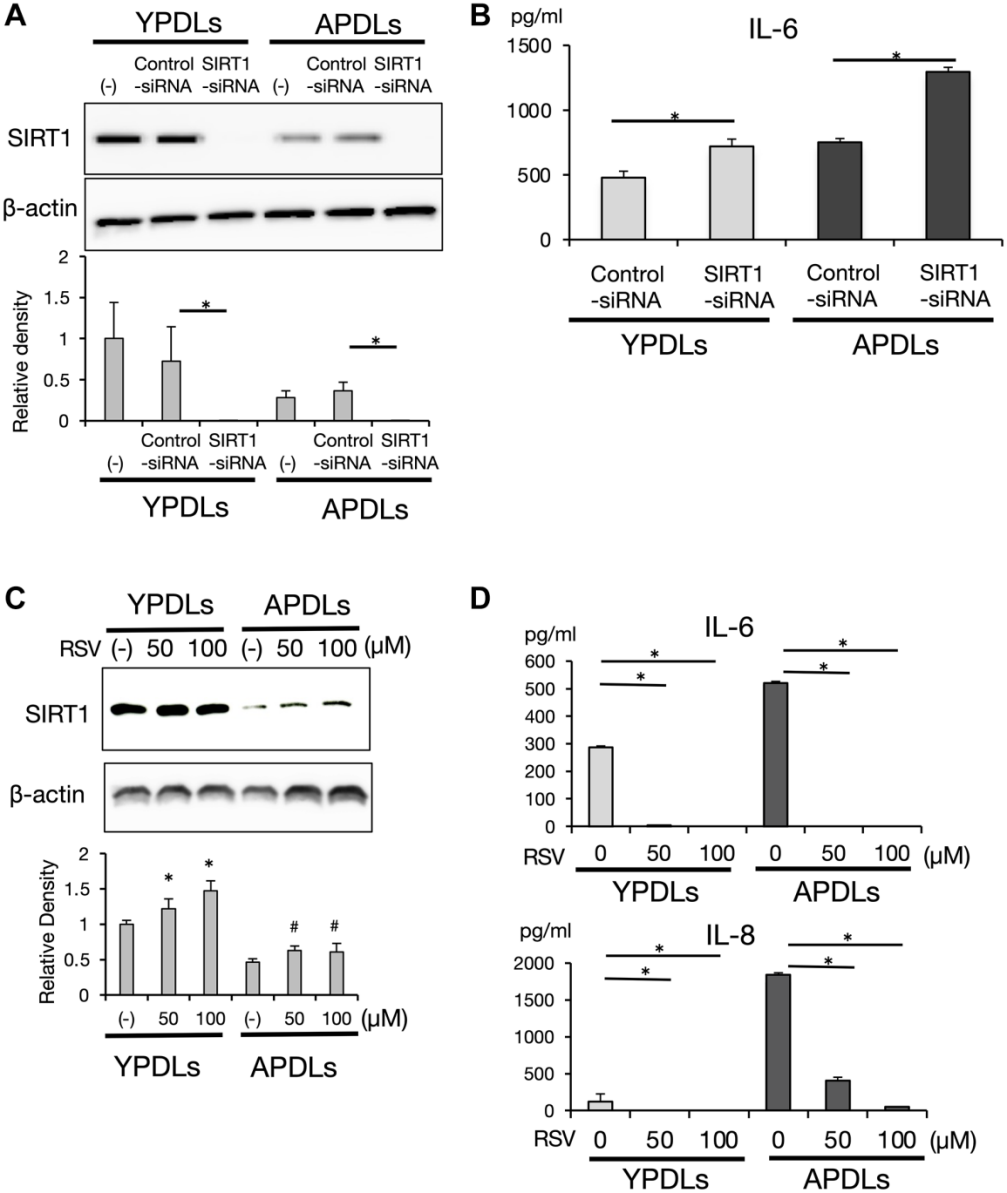
**SIRT1 regulates IL-6 production in senescent HPDL cells.** (**A**) Expression of SIRT1 after si-SIRT1 or si-control transfection into YPDLs and APDLs. Β-Actin was used as a loading control and the relative protein levels were quantified (^*^p < 0.01 vs. control). (**B**) Expression of IL-6 after si-SIRT1 or control transfection into YPDLs and APDLs. IL-6 production from YPDLs and APDLs was measured by an ELISA (^*^p < 0.01 vs. control). (**C**) Expression of SIRT1 after SIRT1 activator, resveratrol treatment, at the protein level measured by western blotting. Β-Actin was used as a loading control and the relative protein levels were quantified (^*^p < 0.01, ^#^*P* < 0.05 vs. none). (**D**) IL-6 and IL-8 productions from YPDLs and APDLs after resveratrol treatment (0, 50 and 100 μM) (^*^p < 0.01 vs. none). Abbreviation: RSV: Resveratrol. Representative data from three experiments are shown.

To confirm our findings, we examined IL-6 production in SIRT1-deficient senescent HPDL cells. SIRT1 expression in YPDLs was higher compared with APDLs. Si-SIRT1 treatment clearly suppressed SIRT1 expression in HPDLs at protein level (Figure 6A). Consistent with this finding, si-SIRT1 treatment significantly enhanced IL-6 production in both of YPDLs and APDLs (*p* < 0.01) (Figure 6B). Treatment with resveratrol (RSV), a polyphenol and well-known activator of SIRT1, slightly increased SIRT1 protein expression (Figure 6C) and inhibited IL-6 and IL-8 production in both of YPDLs and APDLs (*p* < 0.01) (Figure 6D). These results suggest that a sufficient level of SIRT1 activity was required to maintain IL-6 at low levels in HPDL cells.

## DISCUSSION

Physiological roles of cellular senescence in the maintenance of periodontal tissue homeostasis and pathogenesis of periodontitis have not yet been fully elucidated. In this study, we observed many senescent PDL cells in the periodontal tissue of aged mice with apparent alveolar bone loss. The ratio of SA β-gal-positive cells in the periodontium was greater than that in the bone and gingival connective tissue. In addition, we determined miR-34a partly regulated SASP trough the regulation of NF-κB by SIRT1 in HPDL cells. Previous studies reported a rejuvenating effect of rapamycin and a relationship with TLR9 in periodontal aging [6–8]. However, our findings suggest a novel mechanism for the pathology of chronic inflammation in periodontal tissue that is partly mediated by senescent PDL cells with SASP. To the best of our knowledge, this is the first study to identify: 1) the potential for senescent PDL cells to induce inflammation of periodontal tissue, and 2) a miRNA-dependent molecular mechanism of SASP in senescent PDL cells.

Aged mice showed apparent alveolar bone resorption. Notably, bone resorption in aged mice was induced without artificial infection of bacteria such as *P. gingivalis*. SA β-gal-positive senescent cells were found in alveolar bones, periodontal ligament, tooth pulp, and gingival connective tissue. In particular, periodontal ligament showed a large number of SA β-gal-positive cells (Figure 1B). These results suggest that chronological aging accelerates organ senescence in periodontal ligament even without attachment of periodontopathic bacteria. Our results are consistent with these findings, namely the observance of hyposensitivity of APDLs against *P.g.* LPS (Figure 4). Additionally, endothelial cells and perivascular endothelial cells were positive for SA β-gal in periodontal ligament. Accumulation of senescent endothelial cells has been reported in the coronary artery wall of elderly people [34]. Therefore, we think the localization of SA β-gal-positive cells in periodontal ligament suggests similar pathologies of vascular defects, artery infarction, and periodontitis. Recently, cellular senescence was found in early embryogenesis and wound healing processes of mice [44]. We believe the requirement of cellular senescence for the development and maintenance of PDL under physiological conditions, but not the senescence of organs, requires further study. Taken together, our findings suggest fragility points in aged periodontal tissue and could be applied to new prevention methods or periodontal therapies by targeting senescent cells in aging periodontal ligament.

To examine cellular senescence in periodontal ligament *in vivo*, we induced replicative senescence with telomere shortening in HPDL cells by continuous passaging. However, induction of SA β-gal was not achieved in all APDLs (Figure 2B). Previous studies have revealed that SASP proteins act on their originating or neighboring cells to promote cellular senescence in autocrine or paracrine manners [45, 46]. Our findings may suggest that cellular senescence spreads to other cells in an autocrine or paracrine manner via secreted factors or cell-to-cell interactions in aged HPDL cells, in addition to DNA damage. Therefore, we focused on SASP as the pathophysiological factor in chronic inflammation of aged periodontal tissue. Moreover, SASP has been reported to inhibit proliferation of other normal cells and even promote the progression of cancer [47]. These studies are consistent with our findings (Figure 2). Senescent HPDL cells showed production of representative SASP proteins and enzymes (Figure 3). SASP factors in senescent HPDL cells, including inflammatory cytokines, chemokines, and MMPs/TIMPs, may strongly contribute to the inflammation and destruction of aged periodontal tissue. In this study, we did not determine senescent HPDL-specific SASP factors in terms of types and their combinations. Even so, we believe our findings of regular SASP factors in senescent HPDL cells strongly indicate important roles of cellular senescence in the common pathology underlying periodontitis and age-related chronic diseases in aging populations.

Age-dependent inflammation, known as “inflammaging”, is an emerging concept to explain age-dependent inflammation pathology, which is characterized by infiltrated immunocompetent cells and proinflammatory cytokine production at organ or systemic levels [48]. A general feature of aged tissue is low-level chronic inflammation without apparent bacterial infection, which is termed as sterile inflammation [48]. It has been reported that gingival fibroblasts from aged mice show lower IL-6 production after *P.g.* stimulation compared with those from young mice [49]. Consistent with this, our results indicated that chronic inflammation in senescent HPDL cells was acquired in the steady state, and IL-6 production in senescent HPDL cells was enhanced by proinflammatory cytokine stimulation, but not a bacterial pathogen (Figure 4). As one of the causes of the SASP phenotype with hypo-responsiveness to bacterial stimulation in senescent HPDL cells, DNA damage is thought to induce a proinflammatory cytokine signaling cascade via NF-κB and alteration of the IL-1R/Toll-like receptor signaling pathway [8].

To shed light on the molecular mechanisms regulating cellular senescence in HPDL cells, we focused on miRNAs. It has become clear that miRNAs regulate the onset and progression of diseases as well as their development by regulating cell proliferation, differentiation, apoptosis, metabolism and cellular senescence [19, 20]. *Lin-4,* which was the first identified miRNA in *C. elegans*, has been revealed to affect lifespan [50, 51]. To identify specific miRNAs in senescent HPDL, comprehensive miRNA analysis was performed in our study. MiR-146a, which is highly expressed in diseased sites of rheumatoid arthritis and other inflammatory diseases [52, 53], has been reported as a crucial factor for inflammation. IL-6 expression was altered by treatment with synthetic mimic oligos of miR-146a without stimulation by bacterial pathogens in HPDL cells (Supplementary Figure 6); however, endogenous expression of miR-146a was increased with the passage number and its peak expression was later than that of IL-6 and effect of inhibitor of miR-146a was not concordant (Figure 3 and Supplementary Figure 6). Therefore, miR-146a might play roles in terminating the inflammatory cytokine response to maintain the chronic inflammation of senescent HPDL cells. Tumor suppressor p53 induces miR-34a and miR-34a that affect cyclin-dependent kinases CDK4/6, anti-apoptotic BCL2, and longevity gene SIRT1 [54, 55]. Additionally, miR-34a has been reported to regulate reprogramming of somatic cells and expression of longevity gene SIRT1 [56]. In contrast to miR-146a, expression of miR-34a and IL-6 was increased with the number of cell passages in a coordinated manner and their peak expression was synchronized, while SIRT1 expression was decreased with the progression of HPDL cell passaging (Figures 5 and 6). In fact, miR-34a mimic treatment suppressed SIRT1 expression and upregulated NF-κB activity in HPDL cells (Figure 5C, 5E). The results of si-SIRT1 treatment strongly indicate a SIRT1-dependent IL-6 production mechanism in HPDL cells (Figure 6). Although RSV treatment slightly increased SIRT1 and strongly suppressed IL-6 in APDLs, we believe that RSV may suppress IL-6 production through the activation of other SIRT family proteins, mitochondria, and mTOR-dependent pathways, in addition to SIRT1. A recent study reported improvements in the reprogramming efficiency of miR-34a knockout mouse-derived somatic cells with Oct3/4, Klf4, Sox2, and c-Myc. Therefore, miR-34a is thought to play roles in maintenance of stemness, especially in cancer cells [57]. Accordingly, we believe that miR-34a might be important for the development and wound healing of periodontal tissue through effects on PDL stem cells.

Class 1 and 2 HDAC-specific inhibitor TSA at 400 nM induced IL-6 expression in normal HPDL cells. However, TSA is generally effective at nanomolar levels in mammalian cells, and TSA at 200 nM showed no effects (Figure 5F). Therefore, we believe that HDACs other than the Sirtuin protein family, may not be involved in regulation of IL-6 by promoting the acetylation of lysine at the histone in senescent HPDL cells. Taken together, these findings suggest that accumulation of environmental stress triggers activation of the p53-miR-34a axis that suppresses SIRT1 and promotes NF-κB-dependent IL-6 production in senescent HPDL cells.

This study had several limitations. It was designed and mainly carried out using primary HPDL cells *in vitro*. In our study, it was unclear whether and how the SASP of aged HPDL cells enhanced or resolved inflammation with senescent cell clearance by inducing immunocompetent cells *in vivo*. It is conceivable that experimental periodontitis can be induced in an aged animal, which should be examined to confirm the pathophysiological significance of cellular senescence in periodontal ligament *in vivo*. Additionally, to identify causes of senescent cells for the pathophysiology of age-related disease, their elimination has been approached [58–60]. This is an important issue for our study that should be examined in the future. We have been investigated the effects of NMN application in a mouse model of periodontitis and found it to effectively reduce oxidative stress in PDL tissue. To establish the clinical relevance of our study, we intend to perform epidemiological studies combined with analysis of clinical samples, such as extracted teeth with severe periodontitis. At present, we are identifying secreted proteins of gingival crevicular fluids to identify SASP proteins specific to senescent HPDL by proteomic analysis.

In conclusion, cellular senescence may evoke inflammation and destruction of aged periodontal tissues through SASP in senescent PDL cells. Thus, elimination of senescent PDL cells or suppression of the miR-34a-dependent SIRT1-NF-κB axis represents an attractive therapeutic strategy to prevent periodontitis in elderly people. Moreover, our findings may lead to a new area in periodontal medicine-based etiology for age-related systemic diseases. Furthermore, we expect targeting of aging PDL cells to be a bidirectional treatment for complications such as diabetes, which is closely related to periodontal disease.

## MATERIALS AND METHODS

### Reagents

IL-1β (R&D, MN, USA), *P.g.* LPS (WAKO, Tokyo, Japan), *E. coli.* LPS (WAKO) and Rapamycin (WAKO) were applied to cells at the indicated concentrations and periods.

### HPDL cell culture and induction of replicative

Primary human PDL (HPDL) cells (ScienCell Research Laboratories Co., CA, USA) were used in this study. HPDL cells were maintained in α-MEM (WAKO) supplemented with 10% fetal calf serum and antibiotics. To induce replicative senescence in HPDL cells *in vitro*, we used a modified NIH-3T3 cell protocol [61]. Briefly, 1 × 10^6^ HPDL cells were plated on a 10-cm culture dish, cultured for 3 days, then harvested, and counted. Then, 1 × 10^6^ HPDL cells were replated and this passaging cycle was repeated every 3 days. To establish senescent HPDL cells, cell growth of HPDL cells were monitored at each passage. Then, population doublings (PDs) of HPDL cells were calculated by the following formulas [62]:


$${\text{PD}}_{\text{n}}=\log_{2}({\text{Nc}}_{\text{n}}{\text{/Np}}_{\text{n}-1})\text{ and PDs}={\text{PD}}_{1}+{\text{PD}}_{2}+{\text{PD}}_{n}$$

n is passage number, Np_n-1_ is number of plated cells in passage number n-1, and Nc_n_ is the number of collected cells in passage number n.

PDs of HPDL cells were used to draw a growth curve based on the number of proliferated cells at each passage of HPDL cells. PDs of HPDL cells were decreased gradually and the cell cycle was almost arrested at around passaged number (P) 30. In our experimental model, >P30 HPDL cells had decreased cell growth and showed near cell cycle arrest. P30 HPDL cells satisfied the various definitions of cellular senescence, such an enlarged cell shape, high SA β-gal activity, and SAHF formation. We judged that P30 HPDL cells had acquired senescent cell-like characteristics, namely premature senescence. Thus, we used >P30 HPDL cells as senescent or prematurely senescent HPDL cells (APDLs).

### Flow cytometry

Analysis of the cell size and granularity of HPDL cells was performed by flow cytometry (FCM) using a FACSCalibur (BD Bioscience, CA, USA). FSC/SSC values were evaluated by CellQuest™ Pro software (BD Bioscience).

### ROS analysis

To examine the level of intracellular reactive oxygen species (ROS), HPDL cells plated on glass bottom dishes (Matsunami, Tokyo, Japan) were incubated with 2.5 μM CM-H_2_DCFDA (Life Technologies, CA, USA) for ICC staining. FCM analysis was used to evaluate the peak intensity of FL1-H with CM-H_2_DCFDA staining by CellQuest™ Pro software.

### Micro-computed tomography

Young (6-week-old) and aged (68–104-week-old) male C57BL/6 mice were obtained from Japan SLC Inc. (Shizuoka, Japan). The mice were maintained in the Animal Experiment Laboratory of Osaka University Graduate School of Dentistry until the indicated age in experiments. All animal experiments were approved by the Institutional Animal Care and Use Committee of Osaka University Graduate School of dentistry (permit number: 24-012-0) prior to the commencement of experiments. To examine alveolar bone loss in periodontal tissue of mice, microcomputed tomography (μCT) was conducted for quantification. Briefly, alveolar bones including maxillary molars were dissected and observed using an R_mCT2 3D micro X-ray CT system designed for use with laboratory animals (Rigaku, Tokyo, Japan) to evaluate alveolar bone loss. Alveolar bone resorption was measured in CT images using 3D image analysis software TRI/3D-BON (RATOC System Engineering, Tokyo, Japan). Alveolar bone loss was calculated by measuring the distance from the cement-enamel junction (CEJ) of the mesial root (a), the distal root at first molar (b), and the mesial root at the second molar to the alveolar bone crest (c) (Figure 1A–1C).

### SA β-gal staining

Maxillae from mice were fixed in 4% paraformaldehyde (PFA) in phosphate buffered saline (PBS) (Wako) overnight at 4°C and decalcified in 0.5 M EDTA (Wako) for 1 week. After decalcification, periodontal tissues were dehydrated using 15%, 20%, and 25% sucrose in PBS. Then, periodontal tissues were embedded and in O.C.T. Compound (Sakura Finetek, Tokyo, Japan), frozen, and sectioned at 5 μm thicknesses in a mesio–distal orientation using a CM3050 S (Leica Microsystems, Wetzlar, Germany). Senescence-associated β-galactosidase (SA β-gal) activity in lysosomes at pH 5.5–6.0 was examined by a Senescence Detection Kit (Bio Vision, CA, USA) including SA β-gal staining solution, 5-bromo-4-chloroindol-3-yl β-D-galactopyranoside, 5 mM potassium ferrocyanide, 150 mM NaCl, and 2 mM MgCl_2_. For cultured HPDL cells, a slightly modified protocol was applied for SA β-gal staining. Briefly, HPDL cells were washed twice with PBS, fixed in 3% PFA for 3 minutes, washed with PBS, and then incubated overnight in freshly prepared SA β-gal staining solution including 1 mg/ml X-Gal, 5 mM potassium ferrocyanide, 150 mM NaCl, and 2 mM MgCl_2_.

### Immunohistochemical staining

After fixation and decalcification, maxillae were embedded in paraffin blocks (Sakura Finetek, Tokyo, Japan). Tissue samples were sliced from paraffin blocks (4-μm sections) using a REM 710 (Yamato, Saitama, Japan), deparaffinated three times in xylene for 5 min, and hydrated in a methanol gradient (100%, 95%, 70%, and 50%). Blocking of unspecific peroxidase activity was performed for 30 min with 3% H_2_O_2_ and 90% methanol. Target Retrieval Solution [high-pH Citrate buffer (Agilent, CA, USA)] was used for antigen retrieval. The following antibodies were used: p16 (rat anti-CDKN2A/p16INK4a; dilution 1:200; Abcam, Catalog No. ab241543), SIRT1 (dilution 1:200; Abcam, Catalog No. ab189494), Lamin A + Lamin C (dilution 1:200, Abcam, Catalog No. ab133256). Incubation with primary antibody was performed overnight at 4°C. Subsequently, slides were washed with PBS for 10 min. A biotinylated secondary antibody was incubated initially for 30 min, followed by an avidin biotin complex kit (Vector Laboratories, catalog no. BA-4000) for an additional 30 min. VECTSTAIN Elite ABC Reagent (Vector Laboratories, Catalog No. PK-6100) was used for detection. Slides were counterstained with hematoxylin. Antibody-stained cells were counted in the PDL area. Quantification was performed using Image J (National Institutes of Health, Bethesda, MD, USA).

### Confocal fluorescence microscopy

HPDL cells were plated on fibronectin-coated glass coverslips and cultured for 24 hours. Cell layers were fixed in 4% PFA for 10 min, permeabilized with Triton X-100 for 10 min, and blocked with 1.5% BSA for 1 hour. Actin fibers were stained with Anti stain 555 phalloidin or Anti stain 555 phalloidin (Cytoskeleton, CO, USA). Anti-SIRT1 (Cell Signaling Technology; CST, MA, USA) and γH2AX (CST) antibodies were used as primary antibodies and Alexa Fluor 594 goat anti-rabbit IgG (CST) was used as the secondary antibody for ICC staining. Nuclei were stained with VECTASHIELD Mounting Medium with 4'6-diamidino-2-phenylindole (DAPI) (Vector Lab., CA, USA). Immunofluorescence and quantitative image analysis were performed under a Leica SP8 microscope (Leica) using 63× or 100× oil immersion lenses with a numerical aperture (NA) of 1.4. After acquisition, images were processed with Airyscan (Zen software; Carl Zeiss, Oberkochen, Germany).

### Chromatin staining

Nuclei were stained with VECTASHIELD Mounting Medium with DAPI. Formation of senescent-associated heterochromatin foci (SAHF) [63] in HPDL cells was observed by the Zeiss LSM 510 confocal microscope system (Carl Zeiss) with a 100× oil immersion lens with NA 1.4.

### Transmission electron microscopy (TEM)

TEM analysis of HPDL cells was performed on the basis of a previous study [64]. Briefly, HPDL cells cultured on plates were fixed with 2% glutaraldehyde in PBS at 4°C overnight, and then with 2% tetraphosphate osmium at 4°C for 1 hour. Then, HPDL cells were dehydrated with 50%, 70%, 90% and 100% ethanol solutions, embedded in Quetol-812 (Nisshin EM, Tokyo, Japan), and polymerized at 60°C for 48 hours. Ultrathin sections cut enface at 70 nm thicknesses were collected on diamond knifes and placed on copper grids. They were stained with 2% uranyl acetate at R/T for 15 min, and rinsed with distilled water, and then stained with Lead stain solution (Sigma-Aldrich Co., MO, USA) at R/T for 3 min. The grids were observed under a transmission electron microscope (JEM-1400 plus; JEOL Ltd., Tokyo, Japan) at an acceleration voltage of 80 kV. Digital images were captured with a CCD camera (Olympus Soft Imaging Solutions GmbH, Münster, Germany). Slice preparation and imaging analysis were performed in accordance with the protocols of Tokai Electron Microscopy, Inc. (Aichi, Japan).

### Luciferase assay

HPDL cells were transfected with NF-κB reporter constructs (Promega, WI, USA) and microRNA mimic/inhibitor oligonucleotides using Lipofectamine 2000 (Life Technologies). For IL-1β treatment, HPDL cells were treated with IL-1β at 18 h after transfection. PRL-TK was cotransfected for normalization. Cell extracts were prepared at 48 h after transfection and the ratio of *Renilla* to *firefly* luciferase activity was measured using a Dual-Luciferase Reporter Assay System (Promega).

### microRNA array

microRNA array analyses were conducted using the Agilent human miRNA microRNA array (8 × 60 K) miRBase ver 19.0 (Agilent Technologies, CA, USA). cDNA labeling, hybridization, and scanning were performed using the miRNA Microarray System with miRNA Complete Labeling and Hyb Kit and Agilent DNA Microarray scanner (CERI; an Agilent-certified service provider, Tokyo, Japan) in accordance with the manufacturer’s instructions. Hierarchical and K-means cluster analyses using GeneSpring GX 12.0 software (Agilent) were performed to evaluate the miRNA expression profiles of HPDL cells in each passage.

### qRT-PCR

RT-qPCR was performed in accordance with previously described protocols [65]. Total RNA was isolated from cultured cells using a mirVana miRNA isolation kit (Thermo Fisher Scientific, MA, USA) and then converted to cDNA using a High Capacity RNA-to-cDNA Kit (Life Technologies). Semi-quantitative qRT-PCR was performed using the ABI 7300 Fast Real-Time PCR System with Power SYBR Green PCR Master Mix (Life Technologies) and gene-specific primers (TakaraBio, Shiga, Japan) in accordance with the manufacturers’ instructions. Relative expression was determined after normalization to *HPRT* expression. The expression level of mature microRNAs was determined using miScript II RT and miScript SYBR Green PCR Kits (Qiagen, Hilden, Germany) in accordance with the manufacturer’s protocols. *U6* snRNA was used to evaluate mature miRNAs. The PCR primer sequences are listed in the supporting information (Supplementary Table 1). Total RNA was isolated from PDL tissue dissected from extracted molar teeth of mice under stereo microscopy.

### miRNA mimics and inhibitors

miRNA mimics and inhibitors for miR-146a and -34a were purchased from Thermo Fisher Scientific and transduced into HPDL cells on the basis of the manufacturer’s protocols. A miRNA mimic, which is single-stranded locked oligonucleotide, acts as an endogenous miRNA that suppresses target mRNA expression and protein translation. A miRNA inhibitor, a double-stranded locked oligonucleotide, competes with endogenous miRNAs. We used *mir*Vana™ miRNA Mimic Negative Control #1 as a control LNA in accordance with the manufacturer’s protocols, which suggest target gene expression from negative control-transfected samples as a baseline for evaluation of the effect of control and experimental miRNA mimics on target gene expression. Synthetic oligonucleotides were transduced into HPDL cells using Lipofectamine 2000. Then, total RNA, proteins, and culture supernatants of HPDL cells were harvested at 48 h after transfection.

### Western blot analysis

Western blot analysis was performed on the basis of a previous report [65]. Briefly, HPDL cells were lysed in RIPA buffer (Millipore, MA, USA) containing a protease inhibitor cocktail (Roche, IN, USA), 1 mM sodium orthovanadate (Sigma-Aldrich), 1 mM Sodium fluoride, and 10 mM β-glycerophosphoric acid (Wako). Protein concentrations of the lysates were quantified by the Bradford Assay (Bio-Rad, CA, USA). Lysates were denatured in 5× Laemmli buffer containing 2-mercaptoethanol by boiling for 10 min at 95°C. After cooling, the proteins were separated by SDS-PAGE under reducing conditions and transferred to a PVDF membrane (GE Healthcare, IN, USA). Membranes were blocked with 5% dry skim milk for 1 h and then probed with the following primary antibodies: mouse anti-human p53, rabbit anti-human p16, goat anti-human CTGF (Santa Cruz, TX, USA), rabbit anti-human p21, mouse anti-human Rb, rabbit anti-human SIRT1 (CST), and mouse anti-human β-actin (Sigma-Aldrich). After washing, membranes were incubated with secondary antibodies, horseradish peroxidase (HRP)-conjugated rabbit anti-mouse IgG or donkey anti-goat IgG (CST), and visualized with ECL prime Western Blotting Detection Reagents (GE Healthcare).

### Dot blot antibody array

Screening of SASP factors produced by HPDL cells was carried out using a Human cytokine proteome array (R&D Systems, MN, USA) following the manufacturer’s instructions. HPDL cells were passaged every 3 days. Forty-eight hours after cell seeding, the cell culture medium was replaced with fresh medium and 72 hours later, the culture supernatants were harvested for the array. A list of cytokines and chemokines in the dot plot map is showed in the supporting information (Supplementary Table 2).

### ELISA

IL-6 and IL-8 concentrations in HPDL cell culture supernatants were determined using Quantikine Human IL-6 and CXCL/IL-8 kits (R&D Systems) in accordance with the manufacturer’s protocol.

### Statistical analysis

The presented data are representative of all results. All experiments were performed at least three times. Quantitative data are presented as the mean and standard deviation of three assays. Differences between two means were assessed using an unpaired students’ two-tailed *t*-test for two-sample comparisons or one-way analysis of variance for multiple comparisons with the Bonferroni post-hoc test. For statistical comparisons involving more than two groups, a one-way analysis of variance (ANOVA) with Bonferroni post-hoc test or a welch’s *t*-test (for non-parametric data) was performed to determine differences between groups in Box-and-whiskers plots. *P* values of less than 0.05 were considered to indicate significance.

### Data availability

All data supporting the findings of this study are available within the article and its supplementary materials.

## Supplementary Materials

Supplementary Figures

Supplementary Tables
