# Supplementary material for: Cellular senescence with SASP in periodontal ligament cells triggers inflammation in aging periodontal tissue
Source: Aging (Albany NY). 2023 Mar 1;15(5):1279–305. doi: 10.18632/aging.204569 (PMC10042704; doi:10.18632/aging.204569)
Supplement: Supplementary Figures [file aging-15-204569-s001.pdf]

SUPPLEMENTARY FIGURES

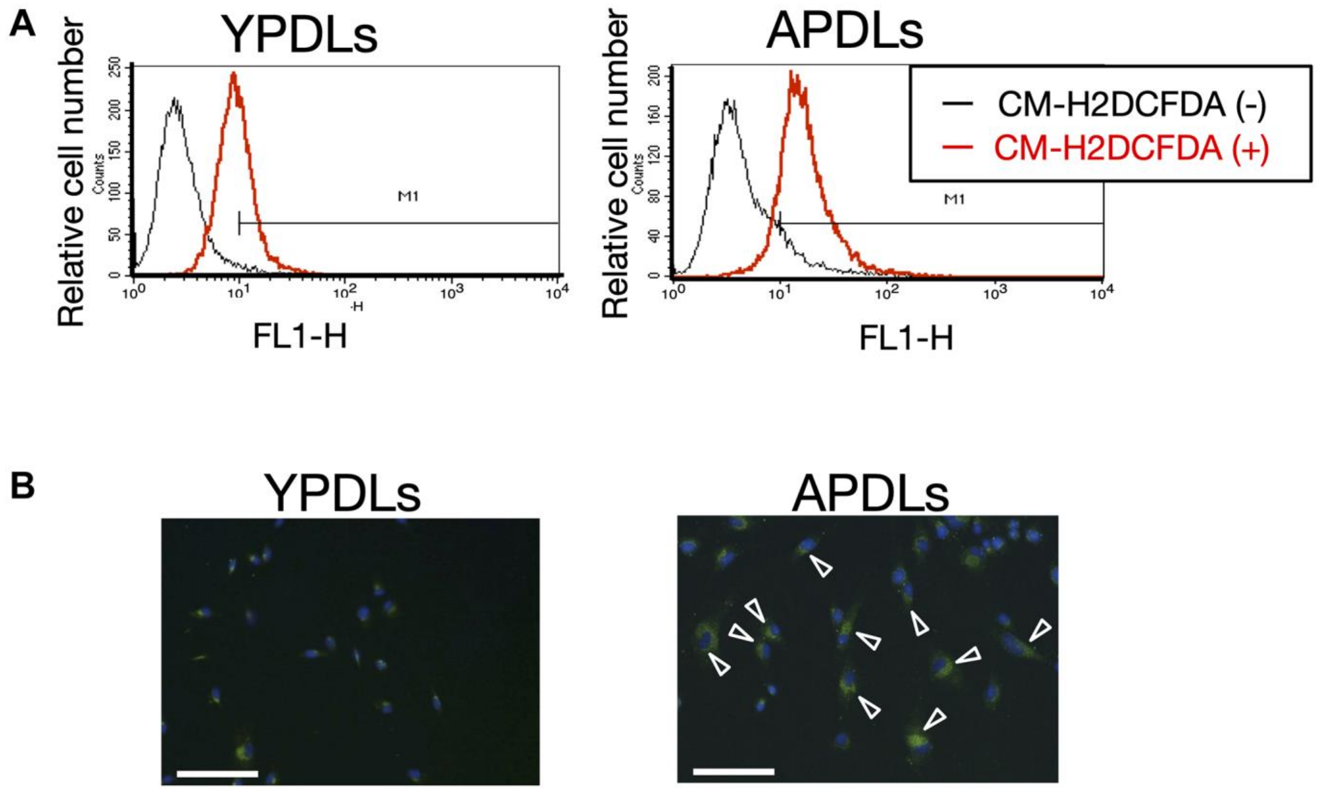

Supplementary Figure 1. Observation of endogenous ROS in YPDLs and APDLs. (**A**) Analysis of cytosolic ROS stained with CF-H2DCFDA in YPDLs and APDLs. The ratio of MFI in control HPDLs to CM-H2DCFDA-labeled HPDLs for aged HPDLs (3.06) was higher than that of young HPDLs (3.16). (**B**) Fluorescence image of cytosolic ROS stained with CF-H2DCFDA in YPDLs and APDLs (1000×). Scale bar = 1 mm.

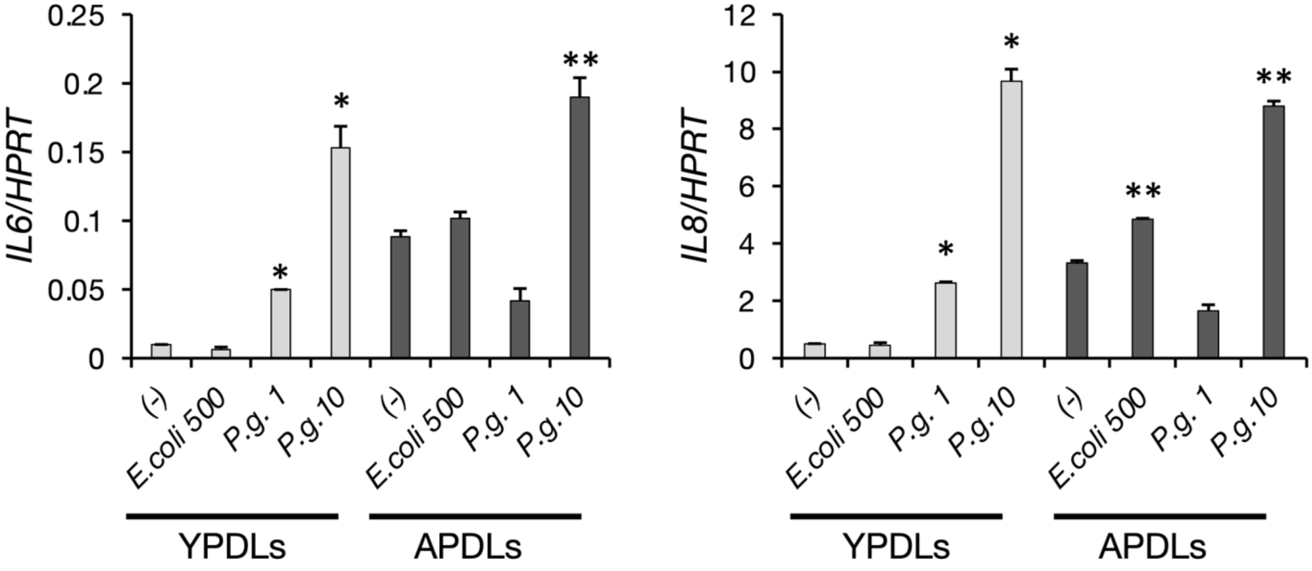

Supplementary Figure 2. Comparison of IL-6 production induced by *P.g* LPS or *E. coli* LPS bacterial pathogens in senescent HPDL cells. Relative mRNA expression of IL-6 following stimulation by *P.g* LPS (1, 10 μg/mL) or *E. coli* LPS (500 ng/mL) in YPDLs and APDLs was quantified by qRT-PCR ($^{*}p < 0.01$ vs. YPDLs none, $^{**}p < 0.01$ vs. APDLs none).

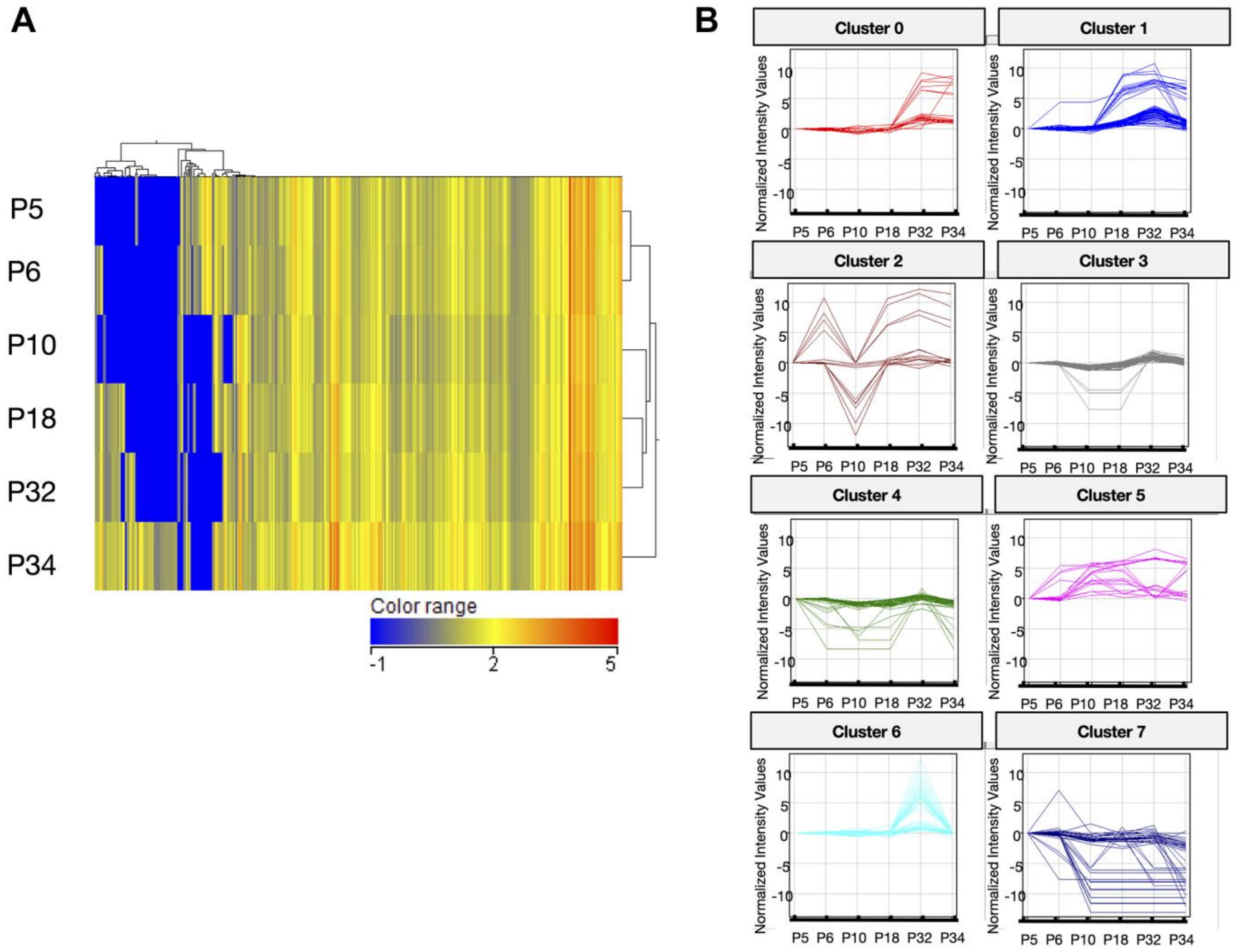

Supplementary Figure 3. Comprehensive analysis of microRNAs in HPDL cells. (**A**) Hierarchical analysis of miRNAs in P5, P6, P10, P18, P32, and P34 HPDL cells. (**B**) *K*-means clustering analysis. The classified eight patterns are shown.

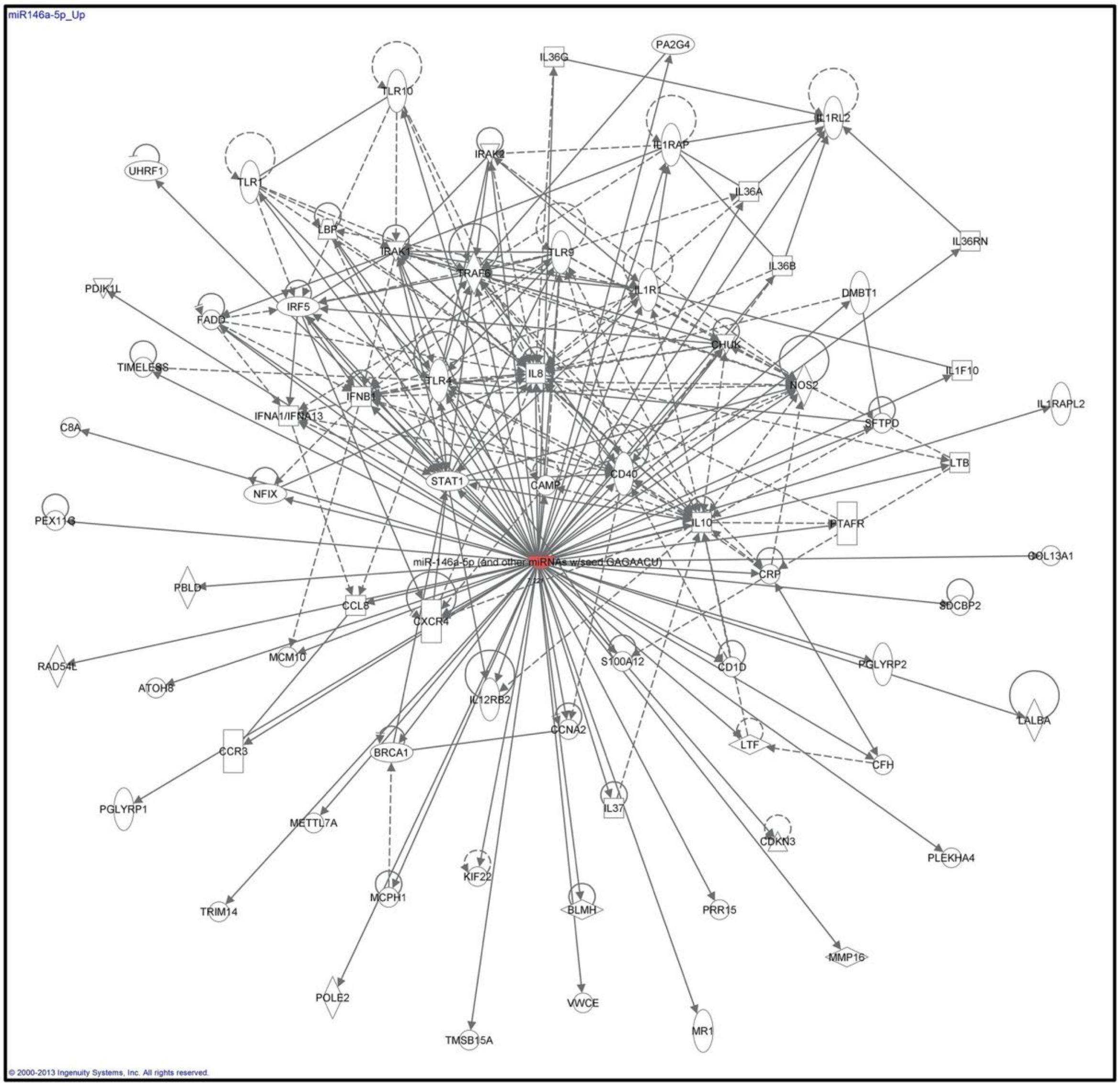

Supplementary Figure 4. IPA analysis of miR-146a in miRNA array analysis.

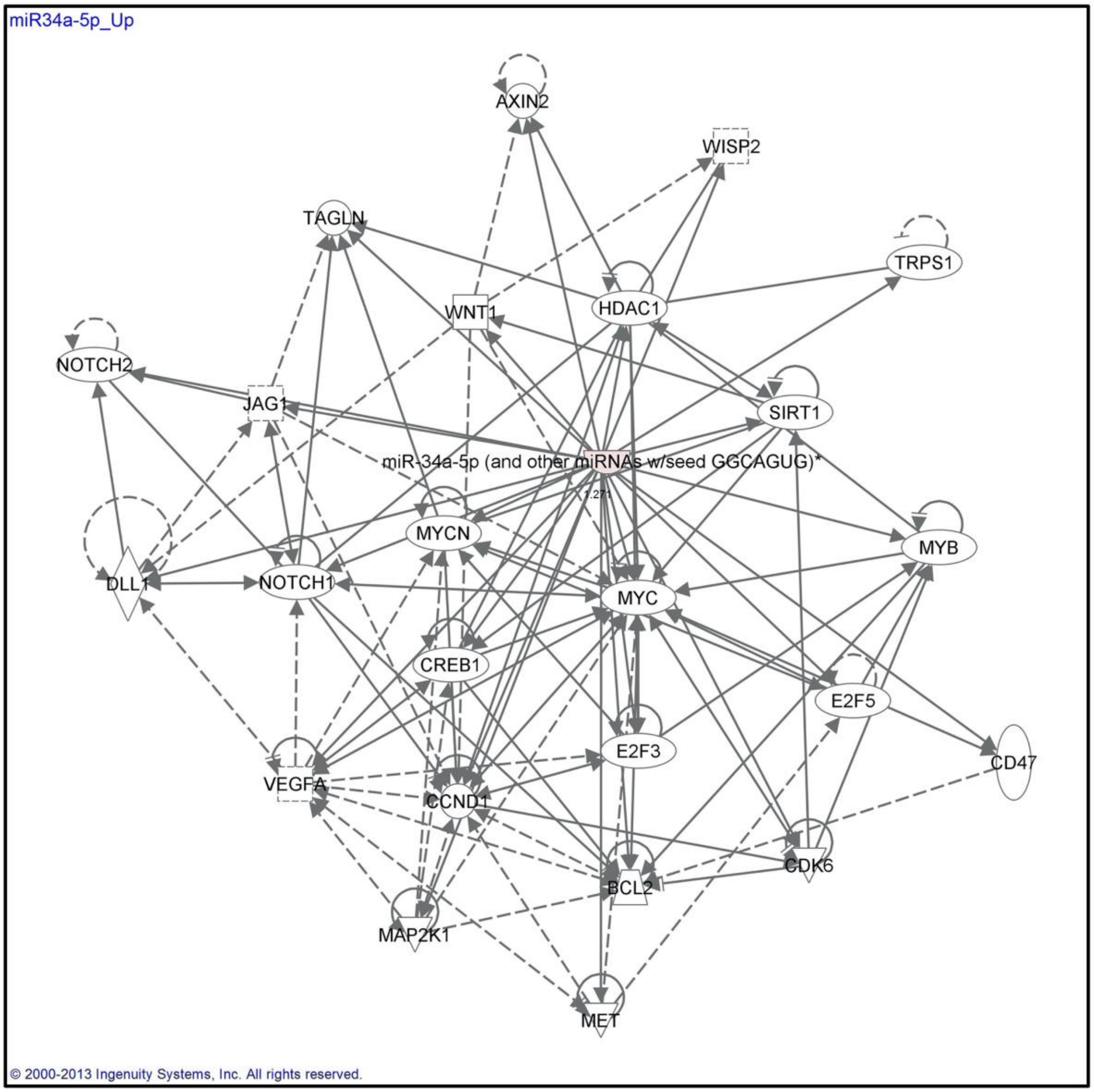

© 2000-2013 Ingenuity Systems, Inc. All rights reserved.

Supplementary Figure 5. IPA analysis of miR-34a in miRNA array analysis.

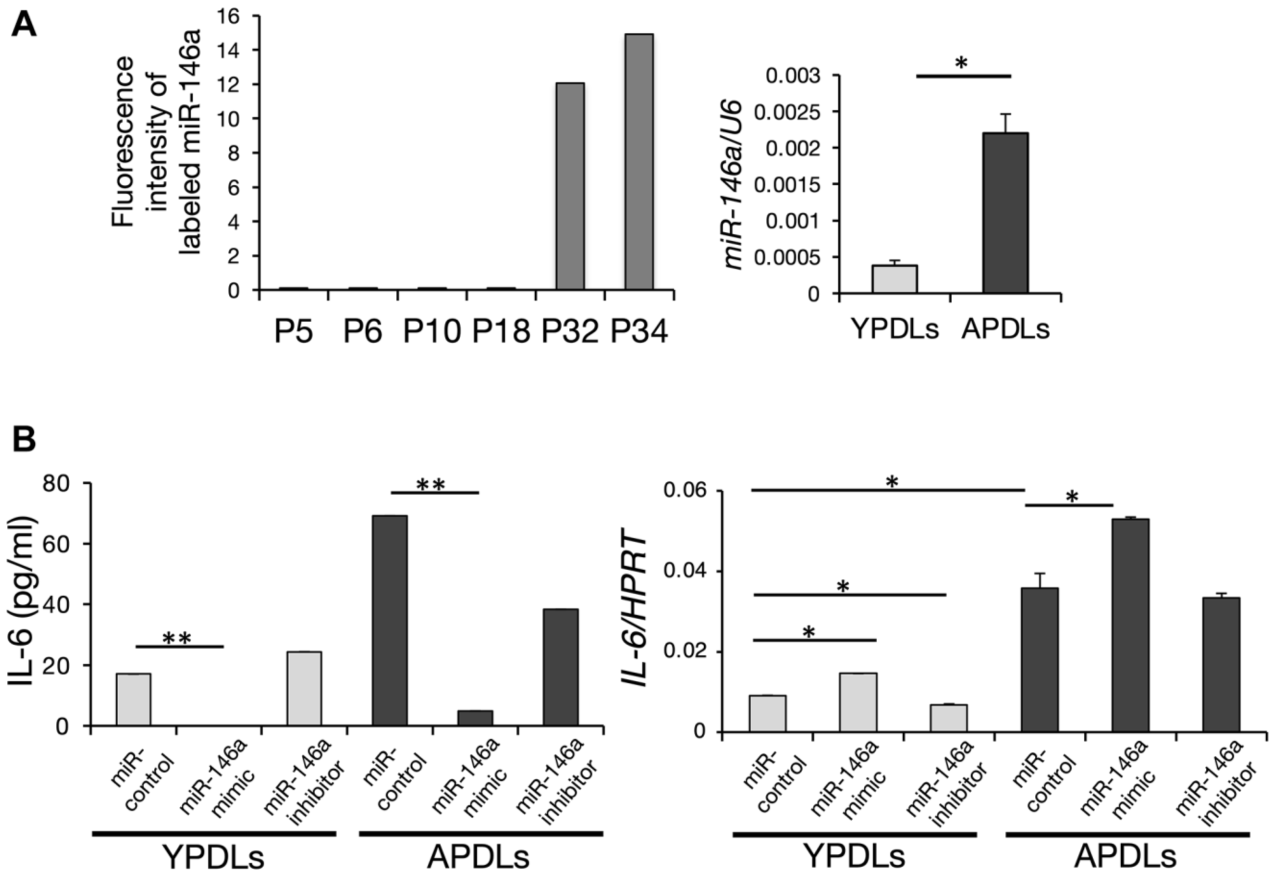

Supplementary Figure 6. Increased expression of miR-146a in senescent HPDL cells. (**A**) Expression of miR-146a was increased depending on the passage of HPDL cells. Scores of the fluorescence intensity of labeled miR-146a in miRNA array analysis of P5, P6, P10, P18, P32, and P34 HPDL cells is displayed in the histogram. Right graph, Expression of miR-146a in YPDLs and APDLs analyzed by qRT-PCR ($^{*}p$ < 0.01 vs. P6). (**B**) Overexpression of miR-146a inhibited IL-6 production in HPDL cells. MiR-146a mimic and anti-miR146a inhibitor oligonucleotides were transfected into YPDLs and APDLs. Expression of IL-6 was measured by an ELISA and qRT-PCR. Representative data from three experiments are shown.
