# Supplementary material for: Cellular senescence with SASP in periodontal ligament cells triggers inflammation in aging periodontal tissue
Source: Aging (Albany NY). 2023 Mar 1;15(5):1279–305. doi: 10.18632/aging.204569 (PMC10042704; doi:10.18632/aging.204569)

SUPPLEMENTARY TABLES

Supplementary Table 1. Primers used in this study.

	human
Gene	**Real time qPCR primers**
P16	5′-CCTTTGGTTATCGCAAGCTG-3′
	5′-CCCTGTAGGACCTTCGGTGA-3′
P21	5′-TCAAATCGTCCAGCGACCTTC-3′
	5′-CATGCCCTGTCCATAGCCTCTAC-3′
TP53	5′-TCGAGATGTTCCGAGAGCTGAAT-3′
	5′-GTCTGAGTCAGGCCCTTCTGTCTT-3′
Klotho	5′-AGCAATCTGGTCTGAATAACACTGG-3′
	5′-CATGTTTCAGCGTGAAAGTTCAAAG-3′
IL6	5′-AAGCCAGAGCTGTGCAGATGAGTA-3′
	5′-TGTCCTGCAGCCACTGGTTC-3′
IL8	5′-ACACTGCGCCAACACAGAAATTA-3′
	5′-TTTGCTTGAAGTTTCACTGGCATC-3′
MMP1	5′-ACAACTGCCAAATGGGCTTGA-3′
	5′-CTGTCCCTGAACAGCCCAGTACTTA-3′
MMP2	5′-CTCATCGCAGATGCCTGGAA-3′
	5′-TTCAGGTAATAGGCACCCTTGAAGA-3′
MMP3	5′-TTTCCAGGGATTGACTCAAAGA-3′
	5′-AAGTGCCCATATTGTGCCTTC-3′
TIMP1	5′-CCTTATACCAGCGTTATGAGATCAA-3′
	5′-AGTGATGTGCAAGAGTCCATCC-3′
TIMP2	5′-GGAGCACTGTGTTTATGCTGGAA-3′
	5′-GACCGAGCGATTGCTCAAGA-3′
SIRT1	5′-CCCAGAACATAGACACGCTGGA-3′
	5′-ATCAGCTGGGCACCTAGGACA-3′
HPRT	5′-GGCAGTATAATCCAAAGATGGTCAA-3′
	5′-GTCAAGGGCATATCCTACAACAAAC-3′
	mice
p16	5′-GACGGGCATAGCTTCAGCTC-3′
	5′-ATTTAGCTCTGCTCTTGGGATTGG-3′
p21	5′-GTCGCTGTCTTGCACTCTGG-3′
	5′-CCAATCTGCGCTTGGAGTGATA-3′
Il6	5′-CCACTTCACAAGTCGGAGGCTTA-3′
	5′-GCAAGTGCATCATCGTTGTTCATAC-3′
Hprt	5′-TTGTTGTTGGATATGCCCTTGACTA-3′
	5′-AGGCAGATGGCCACAGGACTA-3′

Supplementary Table 2. Cytokines and chemokine map for dot plot assay.

A1, A2	A3, A4	A5, A6	A7, A8	A9, A10	A11, A12	A13, A14	A15, A16	A17, A18	A19, A20
Positive Control	C5a	CD40 ligand	G-CSF	GM-CSF	GROα	I-309	sICAM-1	IFN-γ	Positive Control
B1, B2	B3, B4	B5, B6	B7, B8	B9, B10	B11, B12	B13, B14	B15, B16	B17, B18	B19, B20
	IL-1α	IL-1β	IL-1rα	IL-2	IL-4	IL-5	IL-6	IL-8	
C1, C2	C3, C4	C5, C6	C7, C8	C9, C10	C11, C12	C13, C14	C15, C16	C17, C18	C19, C20
	IL-10	IL-12 p70	IL-13	IL-16	IL-17	IL-17E	L-23	IL-27	
D1, D2	D3, D4	D5, D6	D7, D8	D9, D10	D11, D12	D13, D14	D15, D16	D17, D18	D19, D20
	IL-32α	CXCL10/ IP-10	CXCL11/ I-TAC	CCL2/ MCP-1	MIF	CCL3/ MIP-1α	CCL4/ MIP-1β	SerpinE1/ PAI-1	
E1, E2	E3, E4	E5, E6	E7, E8	E9, E10	E11, E12	E13, E14	E15, E16	E17, E18	E19, E20
Positive Control	CCL5/ RANTES	CXCL12/ SDF-1	TNF-α	sTREM-1					Negative Control

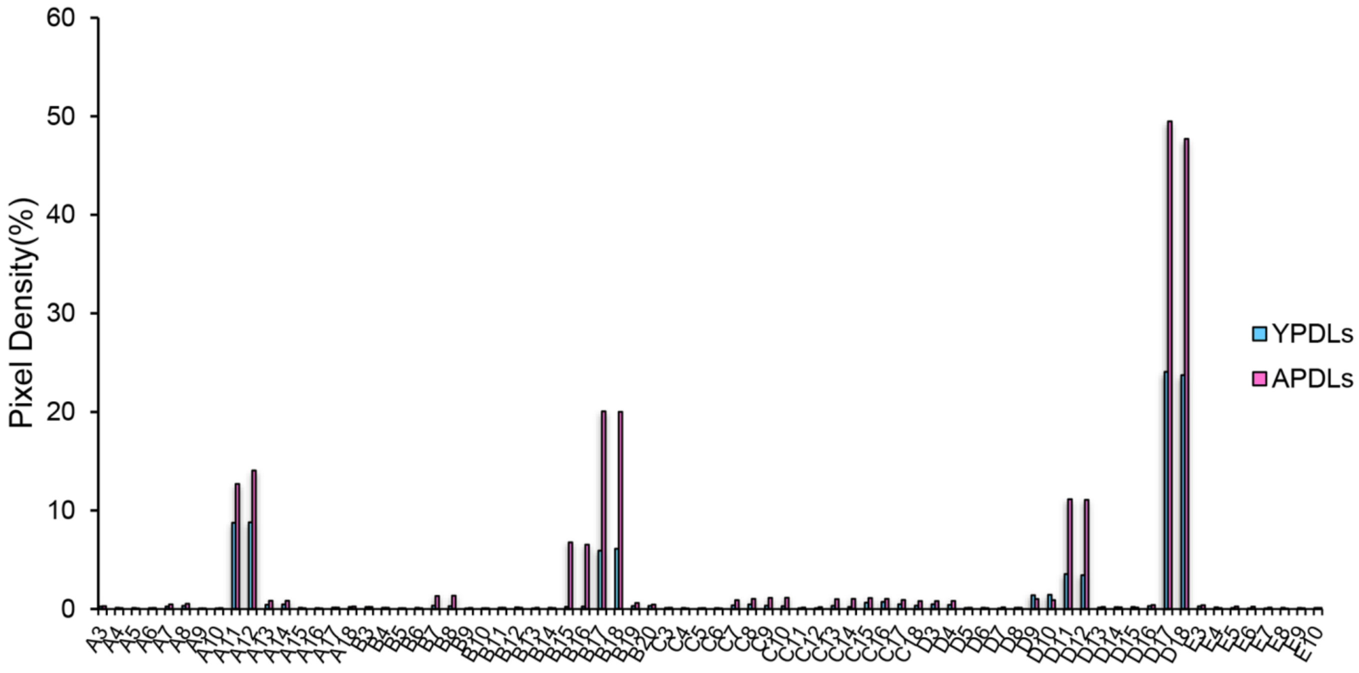

Supplement: Supplementary Tables [file aging-15-204569-s002.pdf]
